# Comparison and Combination of Organic Solvent Nanofiltration and Adsorption Processes: A Mathematical Approach for Mitigation of Active Pharmaceutical Ingredient Losses during Genotoxin Removal

**DOI:** 10.3390/membranes10040073

**Published:** 2020-04-16

**Authors:** Flávio Ferreira, Leonor Resina, Teresa Esteves, Frederico Castelo Ferreira

**Affiliations:** iBB—Institute for Bioengineering and Biosciences, Department of Bioengineering, Instituto Superior Técnico—Universidade de Lisboa, Avenida Rovisco Pais 1, 1049-001 Lisboa, Portugal; flavio.ferreira@tecnico.ulisboa.pt (F.F.); m.leonor.resina@tecnico.ulisboa.pt (L.R.); teresa.esteves@tecnico.ulisboa.pt (T.E.)

**Keywords:** hybrid process, adsorption, API purification, OSN

## Abstract

Active pharmaceutical ingredients (API) are synthesized using highly reactive reagents, catalysts, and solvents. Some of those persist as impurities in the final product and are genotoxic or carcinogenic. The conventional processes used for API purification and isolation are able to achieve the limits imposed by regulatory agencies, but at the expense of significant API losses. Here we report the development of a model to aid in the decision of which dedicated purification process, membrane or adsorption, is most suitable for removal of genotoxic impurities (GTIs), according with a small set of key intrinsic parameters. A hybrid process was developed, combining these two unit operations, to be applied when the use of OSN or adsorption alone result on non-acceptable API losses. Membrane solute rejection and solvent flux was used as parameter for OSN. In the case of adsorption, two isotherm models, Langmuir and Freundlich, were considered. The effect of the recirculation stream and amount of adsorber used on the hybrid process was investigated. Case studies were experimentally validated, confirming that combining the two unit operations can reduce API loss from 24.76% in OSN to 9.76% in a hybrid process. Economic and environmental analyses were performed.

## 1. Introduction

Active pharmaceutical ingredients (APIs) available in the market are mostly synthesized in organic solvent media using highly reactive molecules. Often, low levels of reagents, fractions of catalysts or by-products are present as impurities in the intermediate or final API post-reaction stream. Some of these impurities have unwanted toxicities, including genotoxicity and carcinogenicity. Therefore, related API administration risks for patient’s health caused by the genotoxic impurities (GTIs) has become an increasing concern from pharmaceutical companies and regulatory authorities [1]. Although it is desirable to avoid the use of compounds with genotoxic potential in the manufacture of APIs, this is not always possible. Thus, it is mandatory to ensure that GTI content on the final APIs is controlled below a Threshold of Toxicological Concern (TTC), established by regulatory authorities (1.5 µg/day) [2].

**Figure 1:**
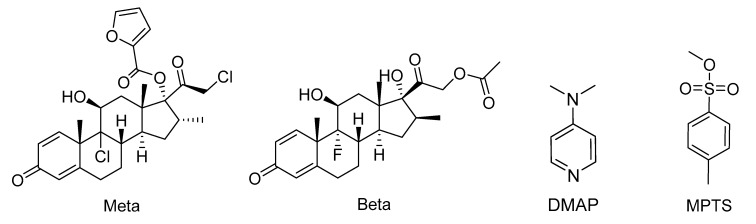
Chemical structure of APIs: Meta and Beta, and potential GTIs: DMAP and MPTS.

In this work, case studies will focus on removal of 4-dimethylamonopyridine (DMAP) and methyl *p*-toluenesulfonate (MPTS) from API solutions, exemplified by mometasone furoate (Meta) and betamethasone acetate (Beta, Figure 1). Although DMAP is not intrinsically genotoxic, after metabolization in vivo, the derived molecules are electrophilic and act as GTIs [3,4]. MPTS is from the sulfonate family of compounds, which are recognized as being DNA alkylating agents [5,6]. Both MPTS and DMAP derived products have the ability to interact with DNA forming DNA-GTI adducts with increased carcinogenic risk. Meta and Beta are corticosteroids used in the treatment of several inflammatory conditions such as asthma and psoriasis, in the case of Meta [7,8], or arthritis, in the case of Beta [9].

There are several strategies in place to mitigate the presence of GTIs in API formulations. However, some are expensive and time consuming (chromatography, solvent exchange), others are not adequate for thermal sensitive APIs (distillation), may lead to high API losses (recrystallization [10,11]) or face challenging working conditions (commercial resins designed to perform in aqueous media, but API synthesis takes place in organic solvents) [5,12,13,14]. Furthermore, none of these techniques targets the selective removal of the impurity, and therefore, losses of the desired product occur [10]. To overcome this, tailor-made adsorbers resorting to molecular imprinting techniques, or incorporation of DNA bases in organic solvent compatible frameworks, to mimic DNA-GTI adduct formation, have been developed to perform in such challenging conditions, but their low adsorption capacity hinders their application at an industrial scale [6,15,16,17,18,19].

Organic Solvent Nanofiltration (OSN) has been suggested, as a low cost and energy technique that allows solute separation ranging from 200 to 2000 Da, coupling solute interaction with membrane facilitated transport, without using high temperatures that may damage thermal sensitive API molecules. For some specific cases, the use of OSN to remove GTIs from API [20] was able to recover API at higher yields and using lower energy than conventional methods, such as chromatography and distillation [21,22].

OSN and polybenzimidazole adsorbers (PBI-TA and PBI-TB, Figure S1) are designed to handle organic solvent solutions based on size discrimination (OSN) or high adsorption capacities (adsorption). In this work both techniques will be explored with a commercial membrane and PBI polymers developed in our group [23].

The use of OSN, in diafiltration mode, removes impurities through the permeate, based in the difference of membrane rejection for retained product and impurities. However, the efficiency of this approach requires a rejection of the product of interest near 100%, otherwise, as diavolumes increase, product losses become significant. Moreover, removal of larger impurities, with higher membrane rejection, implies the use of higher diavolumes. Therefore, the decision on using OSN to achieve the ultralow GTI levels imposed by TTC is not trivial as it can lead to significant API losses which, considering that most of the APIs are very expensive, is usually not economically acceptable. The performance of adsorbers are typically ruled by equilibrium isotherms and, depending on adsorber and solutes, may be indicated for separation of solutes at given concentration ranges.

This study aims to establish a framework to support decisions on the use of OSN, adsorption or combination of those processes to remove GTIs from an API post-reaction stream. A hybrid process, combining OSN and adsorption, was designed to minimize API losses. OSN and PBI adsorbers will be used to validate the theoretical approaches. An economic and environmental analysis will be performed, evaluating these processes using, respectively, economic and green metrics.

## 2. Mathematical Approach: Modelling Section

### 2.1. Set-Up and Boundaries

The three mathematical models established used as **objective function** the value for the GTI/API ratio on the final outlet stream. This ratio was calculated on the basis of the final concentrations obtained (C_out,GTI_ and C_out,API_) on the eluate stream, in the case of adsorption, or retentate, in the case of OSN and hybrid process. The case studies considered a target objective (MaxC) for GTI/API ratio of 7.5 mgGTI/gAPI (Equation (1)):

(1) $$\mathrm{MaxC}=\frac{C_{\mathrm{out},\mathrm{GTI}\;\left(\frac{\mathrm{mg}}{L}\right)}}{C_{\mathrm{out},\mathrm{API}\;\left(\frac{g}{L}\right)}}=7.5\;\left(\frac{\mathrm{mgGTI}}{\mathrm{gAPI}}\right)$$

This value corresponds, for example, to the value required to meet TTC (1.5 µgGTI/day) for API administrations at a dosage of 200 µg/day, as the case previously discussed [10] for airways treatment (e.g., allergic rhinitis and asthma) using the corticoid Meta.

The calculations were performed considering as input a post-reaction solution of 10 g/L of API and 1000 mg/L of GTI. All calculations were based on a processing fixed volume of post-reaction stream. The main operating parameters studied to reach the target objective will be diavolumes (D) and amount of adsorber (m), respectively, for OSN and adsorption operations. The model uses a discrete number of parameters with values within a given range (described below), selected according to previous works [6,10,15,16,23] and/or literature [12,13]. The main parameters considered, for each solute (API and GTI) were membrane rejection and isotherm adsorption constants, respectively, for OSN and adsorption operations. Additionally, membrane solution flux and adsorption kinetics were also considered, in particular on economic analysis, to define membrane areas and operation times.

### 2.2. Organic Solvent Nanodiafiltrations (OSNd)

Membrane rejections of the solutes, the main intrinsic parameter ruling organic solvent nanodiafiltration (OSNd), were computed as a constant parameter along the OSN filtrations according to Equation (2):

(2) $${\mathrm{Rej}}_{x,i}\left(\%\right)=\left(1-\frac{C_{P,x,i}}{C_{F,x,i}}\right)\cdot 100\%$$

where x is GTI or API, C_P,x,i_ and C_F,x,i_ are concentration of GTI or API in the permeate and in the feed, which are variable over diavolume “i” used. C_F,x_, when i = 0, is the concentration of GTI or API fed at the beginning of the diafitration.

Membrane rejections are assumed to be maintained constant over the diafiltrations. The feed volume (V_F_) and retentate volume (V_R_) are maintained constant over diafiltrations and, permeate volume (V_P,i_) and the fresh volume of solvent added (V_Add,i_) is assumed to be equal. The values considered for membrane rejection range between: (i) 0 to 70% for GTI (8 values) and (ii) 80% to 99.99% for API (7 values). Therefore, 56 membranes of different performances were theoretically considered.

Diavolumes, the main operating parameter to be adjusted, are defined as the volume added per initial volume of post-reaction stream:

(3) $$D_{i}=\frac{V_{\mathrm{Add},i}}{V_{F}}=\frac{V_{P,i}}{V_{F}}$$

Based on the assumptions above, the C_R,x,i_ and mass balance equations can be calculated for each diavolume (D_i_) [24] as:

(4) $$\frac{C_{R,x}}{C_{F,x}}=e^{\left[-D_{i}\left(1-{\mathrm{Rej}}_{x}\right)\right]}$$

Applying Equation (4) to GTI and API, and considering the established value of maximum contamination allowed (Equation (1)), one can obtain an algebraic solution for the diavolumes needed for API purification as:

(5) $$D=\frac{\mathrm{Ln}\left(\frac{C_{R,\mathrm{GTI}}/C_{R,\mathrm{API}}}{C_{F,\mathrm{GTI}}/C_{F,\mathrm{API}}}\right)}{{\mathrm{Rej}}_{\mathrm{GTI}}-{\mathrm{Rej}}_{\mathrm{API}}}=\frac{\mathrm{Ln}\left(\mathrm{MaxC}\cdot \frac{{\mathrm{API}}_{\mathrm{in}}}{{\mathrm{GTI}}_{\mathrm{in}}}\right)}{{\mathrm{Rej}}_{\mathrm{GTI}}-{\mathrm{Rej}}_{\mathrm{API}}}=\frac{\mathrm{Ln}\left(7.5\frac{10}{1000}\right)}{{\mathrm{Rej}}_{\mathrm{GTI}}-{\mathrm{Rej}}_{\mathrm{API}}}=\frac{2.59}{{\mathrm{Rej}}_{\mathrm{API}}-{\mathrm{Rej}}_{\mathrm{GTI}}}$$

Note that, in this study, C_R,GTI_/C_R,API_ is the objective function targeted to be 7.5 mgGTI/gAPI in the case studies considered, C_F,GTI_ and C_F,API_ are the GTI and API concentrations on the post-reaction stream to be treated at values of 1000 mg/L and 10 g/L, respectively, i.e., initial concentration contamination was established at a ratio of 100 mgGTI/gAPI.

API losses: The model was used to compute diavolumes required to reach our target objective of 7.5 mgGTI/gAPI, and API losses were calculated by rearrangement of Equation (4):

(6) $$\mathrm{API}\;\mathrm{loss}\;\left(\%\right)=\left\{1-e^{\left[-D\left(1-{\mathrm{Rej}}_{\mathrm{API}}\right)\right]}\right\}\cdot 100\%$$

Operation times: Solution flux through the membrane (J_i_) was also considered as an important parameter for process economics and to define operation times (t_i_) and membrane area required:

(7) $$J_{i}=\frac{V_{P,i}}{A_{m}t_{i}}$$

where t_i_ is filtration time and A_m_ is the area of the membrane. Solution flux through the membrane depends on applied pressure and solution properties that will condition solution permeability through the membrane, such as solvent used, solutes properties and concentration, solution viscosity and resulting osmotic pressure.

### 2.3. Adsorption

The adsorption processes are usually described using isotherm equations. Two isotherm fitting models, namely Langmuir and Freundlich were considered. This study considers that, for a specific adsorber, both GTI and API follow the same adsorption isotherm behaviour and, in the particular case of the Freundlich model, mainly chemisorption takes place with 1/n parameter equal or less than 1. The model does not consider adsorber operation on columns, instead assumes simple adsorber beds operated in batch, fed with API post-reaction stream and unloaded after adsorption equilibrium is reached. The classic equations for Langmuir, Equation (8), and Freundlich, Equation (9), isotherm models and mass balance Equation (10), were considered:

(8) $$q_{e,x,i}=\frac{Q_{\max,x,i}k_{L,x,i}C_{e,x,i}}{\left(1+k_{L,,x,i}C_{e,x,i}\right)}$$

(9) $$q_{e,x,i}=k_{F,x,i}C_{e,x,i}^{\frac{1}{n}}$$

(10) $$V\cdot C_{\mathrm{in},x,i}=V\cdot C_{e,x,i}+q_{e,x,i}\cdot m$$

where q_e,x,i_ (mgGTI/gAdsorber or gAPI/gAdsorber) is the adsorber’s adsorption capacity, Q_max_ (mgGTI/gAdsorber or gAPI/gAdsorber) is the maximum amount of GTI or API bound to the adsorber in a monolayer for the Langmuir model, whereas k_L_ (L/mgGTI or L/gAPI) and k_F_ (mgGTI^1−1/n^/(gAdsorber.L^1/n^) or gAPI^1−1/n^/(gAdsorber.L^1/n^)) are equilibrium constants for the Langmuir and the Freundlich models, respectively, and are related with the energy taken for adsorption, “n” is a parameter related with the surface layer heterogeneity and, V is the volume of the post-reaction stream submitted to the batch adsorption.

Isotherms parameters are the main intrinsic parameters ruling adsorption. While OSN only has one main ruling parameter per solute, each isotherm has two parameters per solute.

For the adsorbers following the Langmuir model, Q_max,x,i_ values were set within the ranges of (i) 1 to 1000 mg/g (four values) for Q_max,GTI,I_ and (ii) 0.0085 to 8.5 g/g (four values) for Q_max,API,i_. Then, for each adsorbent capacity Q_max,x,i_ the solute affinities k_L_ were considered within the ranges of (iii) 0.0081 to 8.1 L/mg for k_L,GTI,I_ and (iv) 0.0021 to 1.1 L/g for k_L,API,i_. The values considered correspond to 16 Langmuir isotherms for GTI removal and other 16 Langmuir isotherms for API binding, resulting in 256 possible combinations of adsorbers theoretically considered with Langmuir isotherm behaviour, from which 160 combinations result on solutions with real numbers.

For the adsorbers following the Freundlich model, parameter n was set for three values: 1, 2 or 3 for both API and GTI and k_F_ values were set within the ranges of (i) 0.05 to 30 mgGTI^1-1/n^/(gAdsorber.L^1/n^) for k_F,GTI_ (11 different values) and (ii) 0.001 to 0.5 gAPI^1−1/n^/(gAdsorber.L^1/n^) for k_F,API_ (6 different values). Therefore, a total combination of i = n × k_L,GTI_ × k_L,API_ = 3 × 11 × 6 = 198 different adsorbers were considered using Freundlich isotherm model, from which 122 resulted on solutions with real numbers.

The adsorber mass, the main operating parameter to be adjusted in an adsorption process, can be calculated algebraically considering our target objective, the isotherms and the mass balance equations for Langmuir isotherms, Equation (11), and Freundlich isotherms, Equations (12)–(14) for *n* equal to 1, 2 or 3, respectively.

(11) $$\begin{array}{c} C_{e,x,i}\\ =\frac{-V-m\cdot Q_{\max}\cdot k_{L,x,i}+k_{L,x,i}\cdot C_{\mathrm{in},x,i}\cdot V}{2k_{L,x,i}\cdot V}\\ +\frac{\sqrt{V^{2}+2m\cdot Q_{\max}\cdot k_{L,x,i}\cdot V+2C_{\mathrm{in},x,i}\cdot k_{L,x,i}\cdot V^{2}-2m\cdot Q_{\max}\cdot C_{\mathrm{in},x,i\cdot }k_{L,x,i}\cdot V+k_{L,x,i}^{2}\cdot C_{\mathrm{in},x,i}^{2}\cdot V^{2}+m^{2}\cdot Q_{\max}^{2}\cdot k_{L,x}^{2}}}{2k_{L,x,i}\cdot V} \end{array}$$

for n = 1, with $C_{e,x,i}=\frac{V\cdot C_{in,x,i}}{k_{F,x,i}\cdot m+V}$:

(12) $$m=V\left(\frac{\mathrm{MaxC}\cdot C_{\mathrm{in},\mathrm{API},i}-C_{\mathrm{in},\mathrm{GTI},i}}{C_{\mathrm{in},\mathrm{GTI},i}\cdot k_{F,\mathrm{API},i}-\mathrm{MaxC}\cdot C_{\mathrm{in},\mathrm{API},i}\cdot k_{F,\mathrm{GTI},i}}\right)$$

for n = 2:

(13) $$C_{e,x,i}=\frac{2C_{\mathrm{in},x,i}\cdot V^{2}+m^{2}\cdot k_{F,x,i}^{2}-\sqrt{4C_{\mathrm{in},x,i}\cdot m^{2}\cdot k_{F,x,i}^{2}\cdot V^{2}+m^{4}\cdot k_{F,x,i}^{4}}}{2V^{2}}$$

for n = 3:

(14) $$C_{e,x,i}=\sqrt[{3}]{\frac{-C_{\mathrm{in},x,i}\cdot m^{3}\cdot k_{F,x,i}^{3}}{2V^{3}}+\sqrt{\frac{C_{\mathrm{in},x,i}^{2}\cdot m^{6}\cdot k_{F,x,i}^{6}}{4V^{6}}+\frac{m^{9}\cdot k_{F,x,i}^{9}}{27V^{9}}}}+\sqrt[{3}]{\frac{-C_{\mathrm{in},x,i}\cdot m^{3}\cdot k_{F,x,i}^{3}}{2V^{3}}-\sqrt{\frac{C_{\mathrm{in},x,i}^{2}\cdot m^{6}\cdot k_{F,x,i}^{6}}{4V^{6}}+\frac{m^{9}\cdot k_{F,x,i}^{9}}{27V^{9}}}}+C_{\mathrm{in},x,i}$$

Note that to meet the objective (MaxC), the variable $C_{e,x,i}\in {\mathbb{R}}_{+}^{*}$. Since polynomial equations, such as quadratic and cubic, assume imaginary numbers as possible solutions, Equations (13) and (14) are the ones that satisfy at the same time the existence conditions for API and GTI (see more details in SI).

The adsorber mass was calculated either algebraically or iteratively, using Equations (12)–(14), for n = 1, 2 or 3, respectively. For iterative calculations, the “solver” mathematical function from Excel, version 2013, was used to obtain the GTI and API concentrations in solution at equilibrium with the adsorber, i.e., C_e,API,i_ and C_e,GTI,i_, and from those values the mgGTI/gAPI ratio was calculated. Such calculations were performed considering 1000 mg/L and 10 g/L of GTI and API initial concentrations and the different parameter values (see range of parameters on isotherms considered) and an arbitrary first value for the mass of adsorber. Then, the calculations of C_e,GTI,i_ and C_e,API,i_, are repeated successively, changing the value of adsorber mass until a value is found to which the calculated API and GTI concentrations meet the target value of 7.5 mgGTI/gAPI. API losses were calculated, using the adsorber amount for each combination of isotherm parameters as:

(15) $$\mathrm{API}\;\mathrm{loss}\;\left(\%\right)=\left[1-\frac{C_{R,\mathrm{API},i}}{C_{\mathrm{in},\mathrm{API}}}\right]\cdot 100\%$$

where C_R,API,i_ was calculated by Equations (13) and (14) for each adsorber mass considered and isotherm constants assumed.

Operation times: Adsorption kinetics must be considered as an important parameter for process economics to define operation times. The operation time must be equal to the time needed to reach the equilibrium concentration (note that, to determine the time of contact between the stream and adsorber, performing a laboratory assay is needed).

### 2.4. Hybrid Process

A hybrid process combining OSN and adsorption was designed to address the cases when OSN or adsorption as single stages are unable to reach TTC value with acceptable API loss.

**Figure 2:**
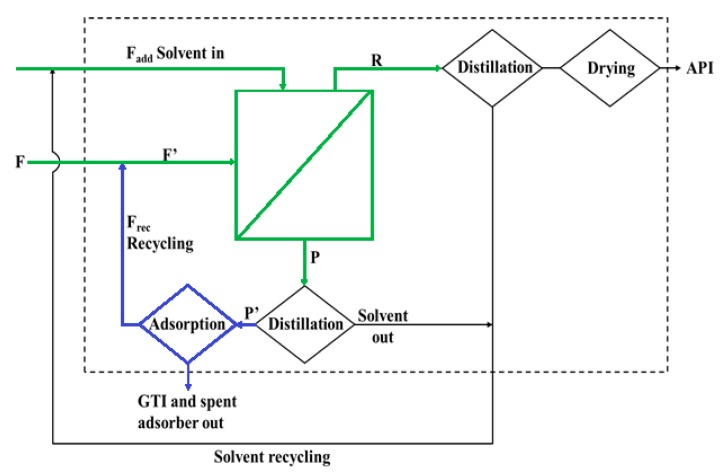
Schematic representation of hybrid process OSN-Adsorption. The dashed line indicates the volume of control for mass balance; in green OSN stage and in blue adsorption stage.

The process is illustrated in Figure 2, composed by three stages:

(i) Diafiltration using an OSN membrane with recovery of purified API in retentate (R)
(ii) Distillation to reduce volume of permeate (P), and
(iii) Adsorption to remove GTIs, i.e., decrease the ratio of C_GTI_/C_API_ for further recirculation of the stream back to feed the next batch OSN stage cycle.

Importantly, the calculations for the hybrid process assume that there are several cycles (indicated by _j_) and calculations are made for consecutive cycles. The set-up of the model and boundaries used are maintained. In other words, a target value of 7.5 mgGTI/gAPI (Equation (1)) is aimed for the OSN retentate (R) stream as target objective and 10 g/L of API and 1000 mg/L of GTI were used, respectively, as C_F,API,i,j_ and C_F,GTI,i,j_ input concentrations for the feed (F) stream for all cycles “_j_”. Note that, the initial ratio of GTI/API of 100 mgGTI/gAPI, should decrease to 7.5 mgGTI/gAPI on the retentate, and consequently will increase to values higher than 100 mgGTI/gAPI on the permeate. Therefore, the adsorption stage offers the route to exit the process and its role is to reduce the GTI/API ratio of the recirculation stream to a value at the level of the feed stream, with minimal API losses. The same volume of feed stream V_F,i,j_ was used for all cycles, from here onwards referred to as V_F_. This model is a simplified approach that does not take into account factors like hydrodynamics or interactions between species.

The same main intrinsic parameters previously considered for the use of OSN or adsorption alone are here used to model the hybrid process, namely, membrane rejection for GTI and API, isotherm constants for GTI and API k_L,x,i_, Q_max,x,i_, k_F,x,i_, n_i_. Again_,_ these parameters were assumed to be constant over operations and several cycles for a given system “i”, (i.e., using a given membrane and adsorber).

The hybrid process was theoretically investigated for selected cases. Such cases correspond to the use of an OSN membrane with rejections of **10%** for GTI and **90%** for API, respectively. For the adsorption stage of the hybrid process, were considered adsorbers that follow either (i) Langmuir isotherms (Q_max_, _API_ = 0.085 gAPI/gAdsorber, k_L_, _API_ = 2.1 L/g and Q_max, GTI_ = 100 mgGTI/gAdsorber, k_L, GTI_ = 0.0081 L/mg) or (ii) Freundlich isotherms (n = 2, k_F,API_ = 0.0078 gAPI^1−1/n^/(gAdsorber.L^1/n^) and k_F,GTI_ = 0.1857 mgGTI^1−1/n^/(gAdsorber.L^1/n^). Note that, to reach the stringent 7.5 mgGTI/gAPI target value using OSN or adsorbers alone, these cases correspond to considerable high API losses estimated, respectively, at values of 27% (using 3.2 diavolume) or 14% (using 94 g/L of Langmuir adsorber) and 99.9% (using 1203 g/L of Freundlich adsorber).

For the hybrid process, the main operating parameters are also still the diavolumes and amount of adsorber.

The amount of adsorber (m_i,j_) was actually maintained constant over the several cycles _j_, and thus from here onwards is referred to as m_i_. To further investigate the effect of this parameter on the hybrid process, the values of adsorber mass considered were 20 g/L or 40 g/L.

The diavolumes (D_i,j_) for OSN, were now calculated for each cycle _j_ by Equation (16) to meet the target value of 7.5 mgGTI/gAPI for each cycle.

(16) $$D_{i,j}=\frac{\mathrm{Ln}\left(\mathrm{MaxC}\frac{{\mathrm{API}}_{\mathrm{in},i,j}}{{\mathrm{GTI}}_{\mathrm{in},i,j}}\right)}{R_{\mathrm{GTI}}-R_{\mathrm{API}}}$$

Moreover, an additional operating parameter, the recirculation/feed streams (Rec/F) ratio, which can be controlled by the concentration factor imposed to the distillation of the permeate (i.e., adjusting V_P’,i,j_/V_P,i,j_ to provide specific R_Rec/F_), was investigated for a range of values between 0.05 to 1:

(17) $$\mathrm{Rec}/F=\frac{V_{\mathrm{Rec},i,j-1}}{V_{F\prime ,i,j}}=\frac{\left(V_{F\prime ,i,j}-V_{F}\right)}{V_{F}}$$

The following stream volumetric balances and equations were established for the hybrid system considering the recirculation loop in each cycle:

(18) $$V_{F\prime ,i,j}=V_{F,i}+V_{\mathrm{Rec},i,j-1}$$

(19) $$V_{\mathrm{Rec},i,j}=V_{P\prime ,i,j}$$

In the diafiltration operation it was assumed (Equations (20) and (21)) constant volume inside the membrane set-up and, diavolumes are established with relation to stream actually fed to OSN (i.e., in relation to F’ and not F) (Equation (22)):

(20) $$V_{F\prime ,i,j}=V_{R,i,j}$$

(21) $$V_{\mathrm{Add},i,j}=V_{P,i,j}$$

(22) $$D_{i,j}=\frac{V_{\mathrm{add},i,j}}{V_{F\prime ,i,j}}=\frac{V_{P,i,j}}{V_{F\prime ,i,j}}$$

For the solutes, x = API or GTI, it is assumed no solute losses on the different process operations. Therefore, the following mass balances were established for feed and the recirculation stream (Equation (23)), membrane operation (Equation (24)), and distillation (Equation (25)):

(23) $$V_{F\prime ,i,j}C_{F\prime ,x,i,j}=V_{F}C_{F,x,i}+V_{\mathrm{Rec},i,j-1}C_{\mathrm{Rec},x,i,j-1}$$

(24) $$V_{F\prime ,i,j}C_{F\prime ,x,i,j}=V_{R,x,i,\mathrm{jF}}C_{R,x,i,j}+V_{P,x,i,j}C_{P,x,i,j}$$

(25) $$V_{P,i,j}C_{P,x,i,j}=V_{P\prime ,i,j}C_{P\prime ,x,i,j}$$

The concentrations on the retentate are as previously calculated according with diavolume and rejection of the membrane for each species:

(26) $$\frac{C_{R,x,i,j}}{C_{F\prime ,x,i,j}}=e^{\left[-D_{i,j}\left(1-R_{x,i}\right)\right]}$$

The concentrations of the solutes, obtained after the adsorption step in the recirculation C_Rec,x,i,j_ can be calculated using V_P’,i,,j_, the C_P’,x,i,,j_ and the Langmuir parameters (Equation (27), adapted from Equation (11)) or Freundlich parameters (Equations (27)–(29) adapted from Equations (12)–(14)):

For Langmuir:

(27) $$C_{\mathrm{Rec},x,i,j}=\frac{-V_{P\prime }-m\cdot Q_{\max}\cdot k_{L,x,i}+k_{L,x,i}\cdot C_{P\prime ,x,i,j}\cdot V_{P\prime }}{2k_{L,x,i}\cdot V_{P\prime }}+\sqrt{\frac{-V_{P\prime ,x,i,j}^{2}+2m\cdot Q_{\max}\cdot k_{L,x,i}\cdot V_{P\prime }+2C_{P\prime ,x,i,j}\cdot k_{L,x,i}\cdot V_{P\prime ,x,i,j}^{2}-2m\cdot Q_{\max}\cdot C_{P\prime ,x,i,j}\cdot k_{L,x,i}\cdot V_{P\prime }+k_{L,x,i}^{2}\cdot C_{P\prime ,x,i,j}^{2}\cdot V_{P\prime ,x,i,j}^{2}+m^{2}\cdot Q_{\max}^{2}\cdot k_{L,x,i}^{2}}{2k_{L,x,i}\cdot V_{P\prime }}}$$

For Freundlich, with n = 1:

(28) $$C_{\mathrm{Rec},x,i,j}=\frac{V_{P\prime }\cdot C_{P\prime ,x,i,j}}{k_{F,x,i}m+V_{P\prime }}$$

For Freundlich, with n = 2:

(29) $$C_{\mathrm{Rec},x,i,j}=\frac{2C_{P\prime ,x,i,j}\cdot V_{P\prime ,x,i,j}^{2}+m_{i}^{2}\cdot k_{F,x,i}^{2}-\sqrt{4C_{P\prime ,x,i,j}\cdot m^{2}\cdot k_{F,x,i}^{2}\cdot V_{P\prime ,x,i,j}^{2}+m^{4}\cdot k_{F,x,i}^{4}}}{2V_{P\prime ,x,i,j}^{2}}$$

For Freundlich, with n = 3:

(30) $$C_{\mathrm{Rec},x,i,j}=\sqrt[{3}]{\frac{-C_{p\prime ,x,i}\cdot m^{3}\cdot k_{F,x,i}^{3}}{2V^{3}}+\sqrt{\frac{C_{p\prime ,x,i,j}^{2}\cdot m^{6}\cdot k_{F,x,i}^{6}}{4V^{6}}+\frac{m^{9}\cdot k_{F,x,i}^{9}}{27V^{9}}}}+\sqrt[{3}]{\frac{-C_{P\prime ,x,i,j}\cdot m^{3}\cdot k_{F,x,i}^{3}}{2V^{3}}-\sqrt{\frac{C_{P\prime ,x,i,j}^{2}\cdot m^{6}\cdot k_{F,x,i}^{6}}{4V^{6}}+\frac{m^{9}\cdot k_{F,x,i}^{9}}{27V^{9}}}}+C_{P\prime ,x,i,j}$$

API losses were calculated as:

(31) $$\mathrm{API}\;\mathrm{loss}\;\left(\%\right)=\left(1-\frac{C_{R,\mathrm{API},i,j}}{C_{F,\mathrm{API}}}\right)\cdot 100\%$$

## 3. Materials and Methods

### 3.1. Materials

4-Dimethylaminopyridine (DMAP) and methyl *p*-toluenesulfonate (MPTS) were purchased from Acros (Merelbeke, Belgium) and were used as supplied, without further purification. Dichloromethane (DCM), methanol (MeOH) and acetonitrile (MeCN) HPLC grade solvents were purchased from Fisher Chemicals (Hampton, NH, USA). Formic acid (FA) was purchased from Panreac (Barcelona, Spain). Mometasone furoate (Meta) and betamethasone acetate (Beta) were kindly provided by Hovione PharmaScience Ltd. (Loures, Portugal). The GMT-oNF-2 membrane was purchased from Borsig Membrane Technology GmbH (Gladbeck, Germany). According with the manufacturer, GMT-oNF-2 is a silicone-based composite membrane stable up to 80 °C and 40 bar in alkanes, aromatics, alcohols, ethers, ketones, and esters, with a molecular weight cut off of 350 Da [25]. Polybenzimidazole adsorbers (PBI-TA and PBI-TB) were prepared as described elsewhere [23].

### 3.2. Apparatus and Analysis

HPLC measurements were performed on a pump coupled to a L-2400 tunable UV detector Merck Hitachi (Tokyo, Japan), using a Nucleosil 100-10 C_18_ reverse phase analytical column (250 × 4.6 mm, Macherey-Nagel, Düren, Germany) an injection volume of 10 µL and the eluents, A: aqueous 0.1% formic acid solution, B: MeCN 0.1% FA solution. For MPTS a flow rate of 2 mL·min^−1^ and UV detection at 230 nm was used; method: 0–12 min, 70%A–30%B. For DMAP, Meta and Beta, UV detection at 280 nm and flow rate of 1 mL·min^−1^ was used with the method: 0–3 min, 60–20% A; 3–4 min, 20% A; 4–8 min, 20–60% A; 8–15 min 60% A. Distillation was performed at atmospheric pressure and 40 °C using a Rotavapor R-3 instrument (Büchi Labortechnik AG, Sulza, Germany).

### 3.3. Organic Solvent Nanofiltration (OSN) Experiments

A dead-end HP 4750 Stirred Cell (Sterlitech, Kent, WA, USA) was used to carry out filtrations of API/GTIs solutions. A pressure of 20 bar was applied using nitrogen, providing the driving force for the filtrations. All experiments were performed under magnetic stirring at 300 rpm. The membrane GMT oNF-2 (Am = 14.6 cm^2^) was preconditioned by filtering pure DCM, until a constant solvent flux was obtained. An HPLC pump Series I (Scientific Systems Inc., State College, PA, USA) was coupled to OSN apparatus and was adjusted to pump fresh DCM at constant flux during the experiment to perform diafiltration. Membrane rejections were estimated using single solute feed solutions of APIs and GTIs at concentrations of 10 g/L and 1000 mg/L, respectively, and solutions of an API contaminated with GTI (ratio 100 mgGTI/g API). Rejection values (Rejx) were calculated from Equation (2) on the basis of solute concentration in feed (C_F,x_) and permeate (C_P,x_).

### 3.4. Adsorption Experiments

Batch binding experiments were performed by placing 50 mg of adsorber in 2 mL Eppendorf vials and addition of 1 mL of 10 g/L of API contaminated solutions with 1000 mg/L of GTI. The suspensions were stirred for 24 h at 200 rpm. After this time the samples were centrifuged, and the supernatant was filtered and analyzed by HPLC for GTI and API quantification. These assays were performed with duplicate samples (note that the values of adsorber mass and concentration of solutions can vary depending on model response). The percentage of GTI or API bound to the adsorber was calculated from Equation (32), where C_in_ (mg/L or g/L) is the initial concentration of GTI or API, and C_e_ (mg/L or g/L) is the final concentration of GTI or API in solution.

(32) $$\mathrm{Binding}\;\left(\%\right)=\left[\frac{\left(C_{\mathrm{in}}-C_{e}\right)}{C_{\mathrm{in}}}\right]\cdot 100\%$$

### 3.5. Binding Adsorption Isotherm Experiments

For the adsorption isotherm experiments at room temperature, 1 mL of DMAP or MPTS solutions prepared in DCM, with different initial concentrations (100, 500, 1000, 2000, 3000, 4000 and 5000 mg/L) were added to 50 mg of the adsorbers (PBI-TA or PBI-TB). The mixtures were stirred at 200 rpm for 24 h. After that time, the suspensions were centrifuged, and the supernatants were filtered and analyzed by HPLC. All experiments were carried out in duplicate. For Meta, solutions with different initial concentrations (100, 500, 1000, 2000, 5000 and 10,000 mg/L) were submitted to the same procedure. The percentage and the amount of GTI or API bound to the adsorbers was calculated from Equations (32) and (33):

(33) $$Q=\frac{V\;\times \left[C_{\mathrm{in}}-C_{e}\right]}{m}$$

where Q (mgGTI/gAdsorber or gAPI/gAdsorber) is the amount of GTI or API bound to the adsorber, C_in_ (mgGTI/L or gAPI/L) is the initial concentration of GTI or API, C_e_ (mgGTI/L or gAPI/L) is the final concentration of GTI or API in solution, V (L) is the volume of solution used and m (g) is the adsorber mass (note that the values of adsorber mass and concentration of solutions may vary depending on model response). The experimental data were fitted to the Langmuir and Freundlich isotherm models [26] according to Equations (8) and (9).

## 4. Results and Discussion

### 4.1. Model Results: Decision Making Framework

Any decision on selection of the API purification route to remove GTIs must consider process efficiency to meet the target TTC value, but also to take into account API losses and operations costs. This study illustrates how to make a decision between the use of diafiltration, adsorption or a hybrid process combining both unit operations. This study starts by a theoretical investigation on the use and optimization of OSN or adsorption unit operations for a wide range of system selectivity. The key operating variable for OSN or adsorption, diavolumes or amount of adsorber, respectively, is first calculated in order to meet TTC values for each system. The results are then discussed in terms of API losses and process solvent intensity. API losses below 10% were considered acceptable, between 10% and 30% not acceptable and more than 30% prohibitive. Then, the hybrid process is studied for selected cases were the OSN or adsorption alone present non-acceptable API losses.

Case studies were first theoretically and then experimentally validated considering solutions of 10 g/L of an API (Meta or Beta) containing 1000 mg/L of a GTI (MPTS or DMAP), i.e., initial 100 mgGTI/gAPI ratio. The API purification of the hybrid process was assessed considering the adsorption properties of PBI-TA or PBI-TB and the permselectivity of a GMT-oNF-2 OSN membrane. A target value for a maximum contamination (MaxC) ratio of 7.5 mgGTI/gAPI was imposed for theoretical and experimental studies.

#### 4.1.1. OSN Diafiltration: Thresholds

The API loss was calculated for each combination of membrane API rejection (ranging from 80 to 99.99%) with a membrane GTI rejection (ranging from 0 to 70%). Each combination of membrane rejection for API and GTI represents a specific case, corresponding to the use of a specific membrane with a different selectivity for a given GTI and API. The diavolume numbers, needed to reach the required ultralow levels of contamination, were first calculated for each case (Table S1). The diavolume number calculated provides a metric for solvent use. The API losses were then calculated (Table 1) for each case, considering the diavolume needed to reach the TTC. The use of higher diavolumes, for the same membrane rejection for API, leads to higher API losses. The use of large volumes of solvent and potential sacrifice of substantial amounts of API, makes not trivial the selection of diafiltration, as an adequate method to remove GTIs.

The API losses were categorized in three ranks. The use of diafiltration, as a single step for API purification was considered acceptable for API losses lower than 10%, non- acceptable when estimated API losses lays between 10% and 30% and of prohibitive use for API losses higher than 30%, respectively, shown in Table 1 in green, yellow and red. The systems for which unacceptable API losses (10% to 30%) are estimated, are actually good candidates to a hybrid process, where API is further recovered from the permeate. However, for systems with API losses higher than 30% (red), most probably the use of diafiltration alone or in combination with other unit operation would not be acceptable, and entirely different separation alternatives, based on solute properties, other than differences on molecular weight, should be sought.

Notice that, acceptable API losses are achieved only for membranes with higher API rejections, which means that for the case considered, with a 100 mgGTI/gAPI ratio used in the feed solution, membranes with API rejections higher than 97.5% should be used. Diavolume numbers used is another important decision criterium as, even if API losses are low, intensive solvent consumption can turn the process impractical from an operation, cost and environmental perspectives. Solvent used can be recycled by distillation, thus for example, considering a solvent recovery rate of 90%, the use of a diavolume of five implies the use of 50% of the volume of the post-reaction stream to be treated. Membranes with rejections above 50% for the GTI require the use of diavolumes higher than 5 (darker colours on Table 1 and grey in Table S1). Therefore, one can suggest using OSN to remove GTIs from API solutions, when there is a membrane able to provide rejections higher than 97.5% for API and below 50% for GTI.

The combination of values on Table 1 can be allocated to real membrane rejections obtained for GTIs and APIs. For example, Szekely et al. selected nine different APIs and 11 GTIs to evaluate the use of OSNd as a single step purification process [20]. Solute rejections were experimentally obtained with GMT-oNF-2 and SolSep membranes at 10 and 20 bar using tetrahydrofuran or methyl ethyl ketone as solvents. By the combination of different API/GTI rejections, five case studies were selected, based on MW of the different species involved: two cases with easy separation (A), two cases with medium separation (B) and, one case with hard separation (C). GTI removal of about 99% was obtained using 5, 2–7 and 5 diavolumes for cases A, B and C, respectively. API losses about 5, 1.8–5.5 and over 20% were determined for cases A, B, and C, respectively. This study did not take into account a specific TTC target value. The model described in this work would be very useful to meet specific separations with minimum API losses. For example, data collected from the literature for OSN using the membrane GMT-oNF-2 in tetrahydrofuran, it is possible to identify 80 combinations of GTI/API where our model could predict diavolumes needed and API losses (see Table S2).

#### 4.1.2. Adsorption: Threshold

Adsorption process can be used to remove GTI content from an API, being described by isotherm models. The same assumptions, concerning initial API and GTI concentrations, respectively, at values of 10 g/L and 1000 mg/L (ratio 100 mgGTI/gAPI) and target level of decontamination of 7.5 mgGTI/gAPI were made for estimation of the amount of adsorber needed. An additional assumption made is that both solutes follow the same type of isotherm, Langmuir or Freundlich, and for Freundlich model with the same “n” value was considered. Additional recovery of API from adsorbers, without GTI desorption, is possible for some adsorption systems [23], but not all, and therefore such option is also not considered in this analysis. Note also that, such additional API recovery step would also imply associated costs.

All mass values presented are calculated based on 1 L of feed solution. Values that imply too high adsorber loads may be of impractical application concerning solid to liquid ratio or be impaired by mass transfer limitations. Conditions requiring more than 15%m/v load of adsorbent (c.a. twice the load experimentally used in the current study) are represented in dark colours on Table 2 and Table 3 (and grey in Tables S4 and S5).

Again, the API losses were classified as acceptable for values lower than 10%, non-acceptable for values between 10% and 30% and prohibitive for values higher than 30%, respectively, shown in green, yellow and red in in Table 2 and Table 3, implying that an adsorber of a different nature or other separation process should be sought. Adsorbers able to remove the GTI to the target value, but at the expense of API losses between 10% and 30% are potential interesting adsorbers to be used on the hybrid process. In this process, most of the API is retained by the membrane and the adsorber is used on the recycle loop to remove GTI from the membrane permeate before it is fed to the membrane unit.

Assuming Langmuir isotherm behaviour for both API and GTI, it is possible to calculate the adsorber mass required to reach the TTC value. The Langmuir’s isotherm for one solute is characterized by two constants (k_L_ and Q_max_), and therefore four parameters are required to model both API and GTI adsorptions and estimate the amount of adsorber required to reach the target value of 7.5 mgGTI/gAPI. Four values were considered for each of the four parameters (see Table S3 for values and nomenclature used), resulting in 256 combinations to cover the region of interest. However, for 96 adsorbers with higher API and lower GTI adsorption capacities, it was not possible to attain solutions in ${\mathbb{R}}_{+}^{*}$ for the ratio of 7.5 mgGTI/gAPI, due to inefficient GTI removal, while significant amounts of API are withdrawn from the solution. The combination of those and conditions with more than 30% API losses represent about half of the region of interest considered on this study, illustrating well the transition region between conditions where adsorption is recommended to remove GTIs to the ones where it is not. Still, it is possible to use adsorption for API purification on conditions with adsorber capacities lower for API and higher for GTI. The value of mass of adsorber required and API losses are presented on Table S4 and Table 2, respectively.

Assuming Freundlich’s isotherm, both API and GTI with the same constant “n”, it is investigated the effect of different values of K_F_ for API and GTI and “n” value concerning the capabilities to remove GTI to reach the TTC value, API losses and needs in terms of adsorber amount. Regardless of the isotherm behaviour, as in diafiltration, the selection of an adequate adsorber must be evaluated based on API losses and the amount of adsorber required. Again, for combinations with equilibria constant higher for API and lower for GTI, depletion of API on the solution and inefficient removal of GTI leads to the impossibility to attain solutions in ${\mathbb{R}}_{+}^{*}$ for the ratio of 7.5 mgGTI/gAPI. The transition regions where adsorption performances suggest (high K_F,GTI_ and lower K_F,API_) the use of this process is well defined for the examples selected. Such transition moves slightly to lower K_API_ for values higher than 1, as higher interactions with API contribute to higher API losses.

Note that, most of the post-reaction streams in the pharmaceutical industry have an organic solvent matrix. Therefore, adsorbers to be used need to be solvent compatible, such is the case of molecularly imprinted polymers (MIPs) and commercial resins reported in the literature for API purification. Szekely et al. developed a MIP with methacrylic acid to perform the removal of 1,3-diisopropyl urea from Meta, roxithromycin or Keppra API solutions, using 100 mg/L of GTI and 10 g/L of API in DCM [19]. Removal of 80% of GTI was achieved with API losses about 15–20% using 50 mgMIP/mL of solution. Kecili et al. studied MPTS (5µg/mL) removal from 21-chloro-diflorasone (500 µg/mL) solutions using several polystyrene–divinylbenzene and silica based scavengers in 2-propanol [12]. The authors obtained 100% GTI removal with silica based Si-Trisamine and macroporous polystyrene–divinylbenzene based MP-Trisamine resin, using 150 mgResin/mL, with no API loss after using 2-propanol:THF (1:1) for API recovery. Lee et al. explored MPTS removal, from solutions in acetonitrile and methanol using 100 mg/L of GTI and 100 mg/L of API [13]. It was possible to remove the GTI completely with API losses lower than 10% using 200 mgResin/mL. The combination of values on Table 2 and Table 3 can be allocated to real adsorption isotherms for different GTIs. Unfortunately, none of the reports described above presented isotherm parameters for API. Moreover, isotherm binding studies of the system API-GTI are scarce in literature, not being possible to make an example of application of our model, as presented done for OSN.

#### 4.1.3. Hybrid Process Calculations

##### Hybrid Process Concept

For many cases, the use of OSN or adsorption alone are unable to reach the TTC value or the use of such single stage processes implies more than 10% API losses. This challenge can be addressed by developing more selective membranes or adsorbers or by further process design. In the OSN process, while the larger fraction of the API is retained by the membrane, a small fraction of API is lost through the permeate, the stream carrying GTI out of the process. A hybrid process is here sought combining an OSN with an adsorption stage, to mitigate API losses (Figure 2). The OSN stage, operated in diafiltration mode, is still responsible to yield a retentate stream with ultralow GTI contents (with a target value of 7.5 mgGTI/gAPI in this study). The permeate stream is concentrated by solvent distillation, submitted to an adsorption stage for removal of GTI and recycled back to the OSN feed of the next process cycle. The use of OSN membranes able to remove the GTI to reach the TTC value, but with the sacrifice of 10% to 30% of the API (at yellow in Table 1) were selected to investigate the use of a hybrid process approach.

When used alone, the adsorption process aims at a higher adsorption of the solute that is actually dissolved at smaller concentrations, the GTI, but at negligible sorption of the solute on higher amount, the API, which is quite challenging. However, when integrated in the hybrid process, a lower performance for the adsorption stage is required. In these three stage processes, the mgGTI/gAPI ratio (see Table 4 for values) fed to the adsorption stage (i.e., the permeate of the OSN, after concentration for distillation) is higher than the one in the post-reaction stream (set at 100 mgGTI/gAPI for this study) and the outlet does not need to reach the ultralow GTI levels (values of 100 mgGTI/gAPI would be acceptable). Moreover, as most of the API is retained by the OSN and only a smaller API fraction is on the recycle loop, any API sorption on the adsorption stage represents a smaller fraction of the total API. Therefore, adsorbers with a low performance on separation of GTI from API on single stage adsorption process can actually perform well when employed on the hybrid process (in blue in Table 2 and Table 3).

In the economic assessment of the hybrid process, one must consider whether mitigation of API losses and consequently on revenue losses, pays off the cost with increases in equipment and operation costs associated to additional purification stages. The lower the API losses in the OSN stage (Table 1), the higher the GTI/API values in the permeate (Table 4). However, for higher GTI/API ratio on the permeate, higher amounts of adsorber are needed, implying higher associated costs. Different combinations of membrane rejections for GTI and API can yield the same API losses and GTI/API ratio as exemplified on Table 4. On such cases, similar performances are required for the adsorption stage, still membrane and distillation operations will have different requests concerning diavolume numbers employed, and thus resulting on different membrane areas, operation time and energy to be accounted in economic and environmental assessment.

The performance of the hybrid process on API losses mitigation is further investigated concerning:

(i) the ratio of the recirculation to feed volumes and
(ii) the amount of adsorber used. The amount of diavolumes is optimized for each cycle, in order to always ensure that the TTC value is met (target value of 7.5 mgGTI/gAPI on the retentate stream).

##### Effect of Recirculation to Feed Stream Ratio V_Rec_/V_F_ to a Fixed Load of Adsorber

**Figure 3:**
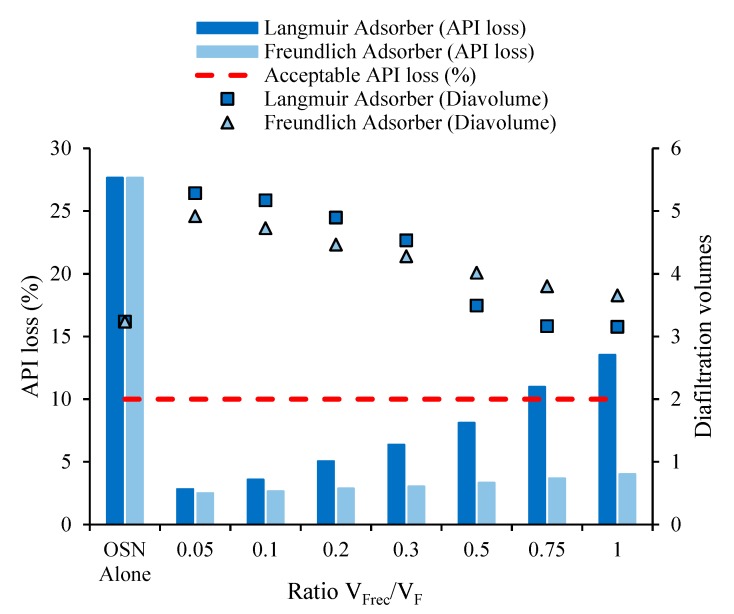
Comparison of effect of ratio recirculation/feed volume on API loss and diavolumes for hybrid process using Langmuir or Freundlich adsorber. Results calculated for membrane rejections of 10% for GTI and 90% for API, and adsorbers that follow either a Langmuir isotherm (Q_max, API_ = 0.085 g/g, k_L, API_ = 2.1 L/g and Q_max, GTI_ = 100 mg/g, k_L, GTI_ = 0.0081 L/mg) or a Freundlich isotherm (n = 2, k_F,API_ = 0.1 gAPI^1−1/n^/(gAdsorber.L ^1/n^) and k_F,GTI_ = 1.5 mgGTI^1−1/n^/(gAdsorber.L^1/n^)).

The volume of recycled stream, i.e., the ratio (V_Rec_/V_F_) of the volumes of recirculated (V_Rec_) to feed stream (V_F_), a crucial variable for the hybrid model, is controlled by the distillation stage. API losses were calculated, after 10 operation cycles, for different V_Rec_/V_F_ ratios using a fixed load of 20 g/L for adsorbers following Langmuir or Freundlich model isotherms (Figure 3).

The V_Rec_/V_F_ ratio impacts directly on the diavolume numbers (and thus process solvent intensity) and on adsorption performance, as different V_Rec_/V_F_ ratio implies different solute concentrations, and thus adsorption at different regions of the solute isotherms (Table S6).

The lower the recirculation volume, the lower the API losses are. However, for low V_Rec_/V_F_ ratio, higher diavolume numbers are needed to meet the TTC value, corresponding to higher solvent and energy intensities. Moreover, too low recirculation volumes may lead to viscous solutions, leading to mass transfer limitations during the adsorption stage. In the hybrid process, as recirculation volume increases, the OSN inlet stream becomes more diluted, requiring a lower number of diavolumes to reach the target value of 7.5 mgGTI/gAPI in the retentate. Still, the volume on the permeate always increases with V_Rec_/V_F_. Therefore, as more API is pushed through the membrane to the adsorption stage, more API is adsorbed (see Table S6), increasing API losses with V_Rec_/V_F_ ratio. Note that, diavolumes are defined on relation to the volume fed to the OSN, V_F_’, i.e., the sum of the volume of post-reaction stream treated (V_F_) with the volume recirculated (V_Rec_). Therefore, the volume added on the diafiltration, that will end up as volume of permeate of the hybrid process, reaches ranges between 1.71 to 1.95 times higher than OSN is used alone, when hybrid process uses Langmuir adsorber, or between 1.60 to 2.26 when it uses a Freundlich adsorber (see Table S7). Importantly, such volumes need then to be concentrated and, from an operational perspective, the bottom fraction of the distillation should not be a negligible fraction. Such a fraction corresponds to the ratio of the recirculate to the permeate stream and it was calculated to be between 1% and 16% (i.e., concentration factors of 100 to 6.6) for the V_Rec_/V_F_ ratio and diavolumes considered (5% for the ratio V_Rec_/V_F_ = 0.3, concentration factor of 20).

Two case studies were investigated using two adsorbers that follow different isotherm behaviours. When used on single stage adsorption process, both of these case studies provide similar performance concerning API losses of 23.85 and 22.94% (in blue on Table 2 and Table 3), but the adsorber with Langmuir behaviour requires a smaller mass (29.81 g/L, blue in Table S4) than the adsorber (82.6 g/L, blue in Table S5) with Freundlich behaviour. The results in Figure 3 were calculated assuming 20 g/L for both cases, but on the context of the hybrid process the GTI concentration fed to the adsorption stage is significantly lower (Table S4). Still, a divergence between these two systems is observed. The streams fed to the adsorption stage become more diluted in GTI and API (Table S6) as the V_Rec_/V_F_ ratio increases, with calculations moving to points of the isotherms where adsorptions of GTI (lower diavolumes) and API (higher API losses) are higher for the Langmuir than for the Freundlich adsorber. Adsorber loads are set as g of adsorber per L of recirculation volume, after distillation, therefore the use of lower V_Rec_/V_F_ ratio also implies the use of lower absolute amounts of adsorber. For the conditions investigated, a V_Rec_/V_F_ ratio of 0.3 was used, as this condition corresponds to API mitigation at acceptable values (bellow 10%), a permeate stream concentration by distillation to 5% of its volume (lower values may show to be impractical), the use of smaller amounts of adsorber and the recirculation stream has API concentrations and GTI/API ratio of low dilution impact on OSN feed solution (Table S6).

##### Effect of Adsorber Amount

**Figure 4:**
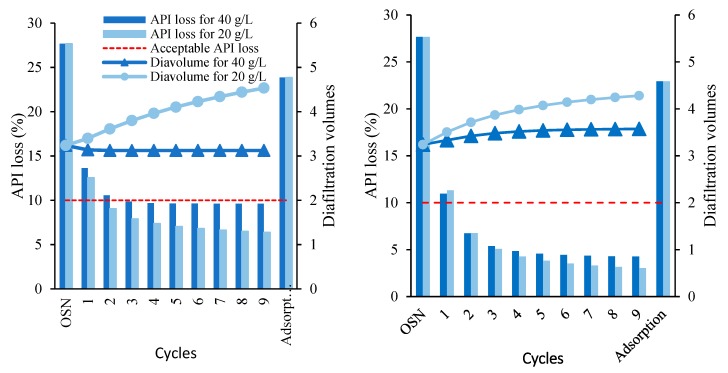
Results for different cycles of the hybrid process combining a OSN membrane with rejections of 90% of API and 10% of GTI at V_Rec_/V_F_ ratio of 0.3 with either 20 g/L (**left**) and 40 g/L (**right**) for an adsorber following Langmuir isotherm (Q_max, API_ = 0.085 gAPI/gAdsorber, k_L, API_ = 2.1 L/g and Q_max, GTI_ = 100 mgGTI/gAdsorber, k_L, GTI_ = 0.0081 L/mg) or Freundlich isotherm (n = 2, k_F,API_ = 0.1 gAPI^1−1/n^/(gAdsorber.L^1/n^) and k_F,GTI_ = 1.5 mgGTI^1−1/n^/(gAdsorber.L^1/n^)). The API losses calculated for the adsorption single stage uses 29.81 and 82.6 g/L of adsorbers following Langmuir and Freundlich isotherms, respectively.

Figure 3 shows the theoretical results for the performance of the hybrid process, after 10 cycles, for different recirculation to feed volume ratio and using a specific membrane and adsorber. Figure 4 shows the results for each of the 10 cycles for hybrid process operated with two different adsorption loads at a fixed ratio V_Rec_/V_F_. The amount of adsorption used is a key parameter as it rules the API losses and GTI removal. The larger the adsorber amount used, the higher the API losses, but also the GTI removal, allowing the OSN stage to operate more efficiently with lower diavolumes. Specifically, the use of 40 g/L instead of 20 g/L at a ratio V_Rec_/V_F_ of 0.3 results on higher API losses, but still below 10%. Moreover, the use of such value as a further advantage to stabilize the number of diavolumes required in the successive cycles, and thus fixing the values for the solvent and energy intensity of each cycle. Additional information on API and GTI concentrations on the stream processes are reported in Tables S6 and S7.

##### Comparison of the Hybrid Process with Other Multi-Stage Processes

Similarly, to the strategy followed by the hybrid process here suggested, several studies developed multi-stage processes to overcome the limitations of specific unit operations. For example, membrane cascades, a configuration for OSN in multi-stages, where the permeate of a previous stage feeds the next stage, enabling the successive recovery of API in each step [27,28]. Peeva et al. demonstrated that, this configuration operated with two stages is able to reach API purifications of 99% from a feed stream of 78% purity, in the case study of DMAP removal from Roxithromycin [29]. Another approach is reported using MIP technology, targeting the increase of purity of API present in OSNd retentate through GTI removal by a highly selective agent [16]. Esteves et al. developed a methacrylic based MIP for DMAP and achieved 99.7% of DMAP removal for a retentate stream containing 100 mg/L of this impurity, with 8% of Meta loss by combining OSNd with MIP-SPE.

OSN has also been described in combination with recrystallization processes to concentrate API in the recrystallization mother liquor as a recycling step. Ferguson et al. demonstrated an increase of Deferasirox API purity from 70% to 98% using OSN to concentrate the API, purging the impurity 4-hydrazinobenzoic acid and recycling the mother liquor back to crystallization [30]. In our group, it was possible to demonstrate API recovery form recrystallization mother liquors using an adsorption stage [10]. In this way, it was possible to minimize Meta loss from 25% to 19% with simultaneous removal of two potential GTIs (DMAP and MPTS) using commercial resins with an improved API yield from 75% to 95.25%.

In the present study, the combination of OSNd with an adsorption stage is suggested in a hybrid approach aiming the recovery of API lost in OSN permeate. To the best of our knowledge is here reported for the first time, the development of a mathematical model to be used as a powerful tool to guide in the choice of the most suitable process for API purification, between OSNd, adsorption or hybrid strategy.

### 4.2. Experimental Assessment

Specific case studies were selected to illustrate experimentally the performance, first of using OSN or adsorption alone, and then of the hybrid process. The chemical structure of the APIs and the GTIs selected for this experimental study are shown in Figure 1. Interestingly, the experimentally estimated values for main parameters for the OSN and adsorption are different when taken from assays made with single solutes or when API and GTI are mixed in the same solution.

#### 4.2.1. OSN diafiltration

**Figure 5:**
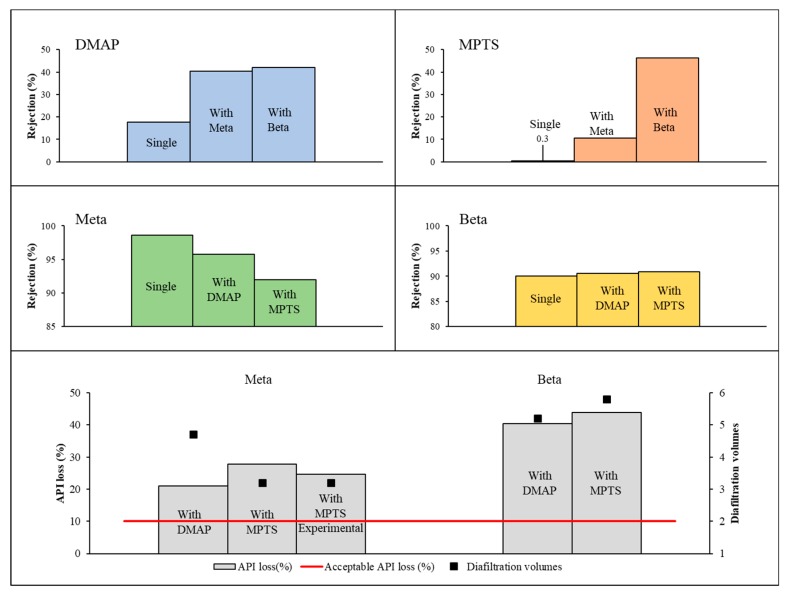
**Top left:** DMAP rejection isolated and in presence of Meta or Beta. **Top right:** MPTS rejection isolated and in presence of Meta or Beta. **Middle left:** Meta rejection isolated and in presence of DMAP or MPTS. **Middle right:** Beta rejection isolated and in presence of DMAP or MPTS. **Bottom:** Comparison between API loss and diafiltration volumes in OSN for Meta and Beta purification.

The rejection of each single compound, and an interaction of one API with one GTI, were assessed experimentally in DCM using a GMT-oNF-2 membrane (14.6 cm^2^) at 20 bar, with flux of 82.13 L·h^−1^·m^−2^. Membrane rejections were estimated experimentally (Figure 5) for the APIs and GTIs used in this study. Membrane rejection for Meta alone was 99%, but reduced to 95% or 90% when mixed with DMAP or MPTS, respectively. On the other hand, the membrane rejections for Beta or Beta mixed with either of the GTIs was always similar, at a value of 90%. Membrane rejection for GTI increases when mixed with API. The membrane rejection for single solute DMAP solutions is 17.66%, but 40.4% and 42.1%, when dissolved with Meta and Beta, respectively. MPTS rejection by the membrane is virtually zero when alone, but it has a dramatic increase to 10.5% and 46.2% when in solution together with Meta and Beta, respectively.

While the lower membrane rejections to API implies higher API loss in the purification process, the increases in GTI rejection implies the need to increase the number of diavolumes, thus using a great amount of solvent and consequently pushing more API to the permeate.

The diavolume number, and the respective API losses, were calculated to reach the target value of 7.5 mgGTI/gAPI (Figure 5, Bottom). Regarding the purification of the selected APIs, in the case of Beta, the membrane has higher rejections to both GTIs (40% to 50%), requiring 5.2 to 6.5 diavolumes to reach the target value of 7.5 mgGTI/gAPI, leading to unacceptable API losses at a value higher than 40%, making the use of OSN alone not a suitable process to purify Beta. In the case of Meta purification, membrane rejection for DMAP and MPTS reaching approximately 40% and 21%, require 4.7 and 3.2 diavolumes to remove GTI to the target value, respectively. Therefore, it is forecast 21.0% or 27.7% of Meta being lost when removing DMAP or MPTS, respectively. In both cases, the use of OSN alone reaches the TTC target value needed but implies a considerable API loss higher than 10%. The calculated values for the four cases studied are presented on Figure 5. The case of separation of MPTS from Meta was assessed experimentally reaching 7.25 mgMPTS/gMeta and 24.73% of Meta losses, with the results matching well the calculated values.

#### 4.2.2. Adsorption

Polybenzimidazole (PBI) was selected as an adsorber for this study. PBI is an organic solvent compatible polymer and we established, in previous work [23], that this polymer, after thermic and acid or alkaline treatment, presents high performance to remove GTIs from organic solvents. Therefore, PBI was submitted to a thermal and acid (PBI-TA) or alkaline (PBI-TA) treatment and used as adsorber to remove GTIs from DCM solutions. Their preparation and isotherms for potential GTIs and API have been reported previously [23], using Meta and Beta as model APIs and DMAP and MPTS as model GTIs.

**Figure 6:**
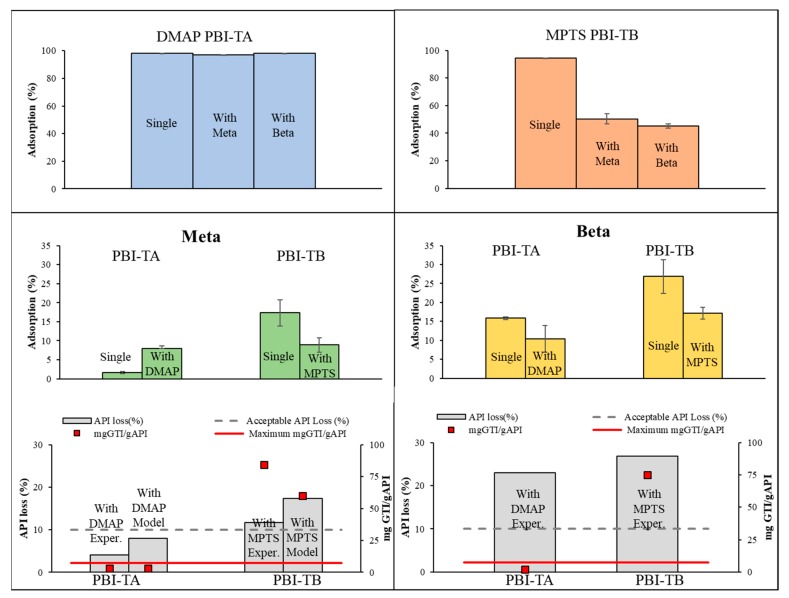
**Top:** Comparison of adsorption of DMAP in PBI-TA (left - blue) and MPTS in PBI-TB (right -orange) for single solute and in API mixtures. **Middle:** Comparison of adsorption of Meta (left -green) and Beta (right -yellow) for single solute and in mixtures with DMAP on PBI-TA or MPTS on PBI-TB. **Bottom left:** Comparison of experimental and theoretical API losses (grey bars) and mgGTI/gAPI reached (red dots) for removal of GTIs from Meta solutions using 50 g/L adsorber. **Bottom right:** Experimental API losses (grey bars) and mgGTI/gAPI reached (red dots) for removal of GTIs from Beta solutions using 50g/L of adsorber. The red line represents the target value of 7.5 mgGTI/gAPI.

The interactions between DMAP and PBI-TA are mainly based on hydrogen bonds and ionic interactions, which are weaker and reversible. MPTS interactions with PBI-TB are also potentially from covalent nature. To illustrate the possible interactions between solutes and adsorbers, the adsorption of single solutes and mixtures of API and GTI were carried out experimentally (Figure 6). While removal of DMAP from an API solution by adsorption on PBI-TA at a value of 98.04% is virtually not affected by the presence of Meta or Beta, the removal of MPTS by adsorption on PBI-TB is reduced from 94.26% to 50.39% and 45.13% on the presence of Meta or Beta, respectively. One possible reason can be the stereochemical impediment caused by API molecules preventing GTI molecules from reaching binding sites of the adsorber, with higher impact on MPTS due to the nature of its adsorption mechanism. Meta and Beta adsorption is also affected by the presence of the GTIs, with a slight increase on their adsorption.

Adsorption on PBI-TA of Meta and DMAP follows a Langmuir adsorption behaviour (Meta: K_L_ = 2.2, Qm = 8.2 × 10^−3^ and DMAP: k_L_ = 8.1 × 10^−3^, Qm = 100, combination A4cI), and Meta and MPTS adsorption on PBI-TB follows Freundlich adsorption behaviour (Meta: k_F_ = 7.8 × 10^−3^, n ≈ 2, MPTS: k_F_ = 0.1857, n ≈ 2). Adsorption on PBI-TA of Beta follows a Freundlich behaviour (k_F_ = 1.5 × 10^−2^, n ≈ 2) and a multi-stage on PBI-TB. Based on the model previously established, the amount of adsorber could be calculated for Meta-DMAP system on PBI-TA at a value of 24.83 g/L. The constants for Meta-MPTS system on PBI-TB, as API is adsorbed on significant amounts along with GTI adsorbed, were not able to reach MaxC of 7.5 mgGTI/gAPI returning an indeterminate form (n.d. value in Table S5). The Beta-GTI system, on PBI-TA and PBI-TB, has different isotherm behaviours depending on the target GTIs used, and the derivation of models for such cases is out of the scope of the current study. Therefore, for direct experimental comparison, a value of 50 g/L was chosen to compare the responses of all the four case-studies in terms of API losses and mgGTI/gAPI reached in the adsorption process (Figure 6, Bottom).

The results show that the use of adsorption alone is efficient to separate DMAP from Meta, reaching experimentally 2.95 mgDMAP/gMeta (model value 3.15 mgDMAP/gMeta) and 8.01% of API loss (theoretical value 4.04%). There is a good fit between the values calculated by the model and the ones obtained experimentally for the mgDMAP/gMeta, but a higher API loss is observed experimentally. Note that, according with the model, only 24.83 g/L of adsorber would be required to reach the 7.5 mgGTI/gAPI threshold, but 50 g/L was experimentally used for comparative purposes, leading to higher removals of GTI (and API) than required.

The experimental results, also confirmed from output values taken from the model, show that, the removal of MPTS from a Meta solution using PBI-TB does not allow to reach the target GTI/API value resulting on high API losses (Figure 6 bottom right). For this case, high differences between calculated (17.32% API losses and 60 mgGTI/gAPI) and experimental values (11.6% API losses, 84.42 mgGTI/gAPI) were obtained. These differences can result from imposing a fit to an integer value of 2 to “n” constant (a better fit would be achieved with n ≈ 1.5).

The experimental result for Beta purification (Figure 6 bottom-right) shows that adsorption can be effectively used to remove DMAP down to 2.18 mgGTI/gAPI using PBI-TA but at the expenses of 23% of API losses. However, this separation could be further optimized, in terms of API losses, decreasing the amount of adsorber, as the use of 50 gAdsorber/L leads to a mgGTI/gAPI value well below the target value of 7.5. For Beta-MPTS system the use of PBI-TB was not efficient as GTI/API values in the end of the adsorption were above the 7.5 mgGTI/gAPI threshold and Beta losses were significant.

In previous works, the process limitations imposed by high API adsorptions to PBI-TB and PBI-TA were addressed by developing an API recovery step. Such step is applied after the GTI removal step and allows to elute API out of the adsorber without minimal elution of the GTI. For the particular case studies here investigated, the application of this step allowed to recover almost 100% of API bound from PBI-TB or PBI-TA, respectively, with virtually no MPTS or 1% for DMAP back contamination [23]. Still, the possibility to retrieve product from the adsorber after the adsorption step, without impurity back contamination, is a very particular case. Therefore, considering the broader scope of the current study and aiming at a general illustration of the single stage adsorption process or of the suggested hybrid process, API recovery step is not considered on this analysis.

As discussed in the previous section, API losses using OSN alone were estimated to be more than 40% and between 20–30% for Beta and Meta purification. Therefore, the case study of Meta will be used to investigate the hybrid process. DMAP can also be efficiently removed from Meta solutions, reaching the TTC, using OSN alone, but at expenses of 21% API losses. The use of PBI-TA adsorption to make this separation, is more efficient, with less than 10% losses on Meta, and therefore for this case study the use of adsorption alone should be considered. The two different processes, OSN and adsorption, have different costs, therefore the economic balance between different API costs and such processes costs are the first cases to be evaluated in the economic analysis.

#### 4.2.3. Hybrid Process

MPTS removal from a Meta solution, to reach the target value of 7.5 mgMPTS/gAPI, using OSN alone implies Meta losses of 25% (experimental values). On the other hand, to reach the low TTC value by MPTS adsorption, requires impractical high amounts of PBI-TB, making interesting to investigate whether the use of this adsorber on the context of the hybrid process would be useful. Therefore, to assess experimentally the hybrid process, we considered solutions containing 10 g/L of Meta contaminated with 1000 mg/L of MPTS in DCM, the use of a GMT-oNF-2 membrane with rejections of 90% for Meta and 10% for MPTS and PBI-TB adsorber (Meta: K_F_ = 0.0078 gAPI^1−1/n^/(gAdsorber.L ^1/n^) and n ≈ 2; MPTS: K_F_ = 0.1857 mgGTI^1−1/n^/gAdsorber.L ^1/n^) and n ≈ 2).

Again, neither OSN nor adsorption alone could practically be used to meet the 7.5 mgMPTS/gMeta. OSN leads to the unacceptable API loss of around 28%, and adsorption stage requires 1203.4 g/L of PBI-TB (which is unpractical) and will lead to 91.59% API losses. The hybrid process was modelled to meet the target value of 7.5 mgGTI/gAPI in the retentate stream. The calculations were performed for optimal values of 3.2 diavolumes, a V_Rec_/V_F_ ratio of 0.3 (implying a concentration factor of 20 for the permeate stream) and the use of 76 g/L of adsorber to remove GTI from the recycling stream. Using these estimations, the process was operated at bench scale for three cycles, being the first cycle loaded with 50 mL of feed and the others with 65 mL (50 mL of fresh solution and 15 mL recycled, ratio V_Rec_/V_F_ = 0.3). The calculated and experimentally obtained API losses and the ratio of mgGTI/gAPI at the retentate are presented in Table 5.

The observed differences between the values predicted by the model and the obtained experimentally are not statistically significant (*p* = 0.43 for API loss and *p* = 0.11 for GTI/API ratio) and can be attributed to rounding values of diavolumes and/or adsorption constants.

For the case study of removing MPTS from a Meta solution, the use of OSN alone using a GMT-oNF-2 membrane results in about 25% Meta losses and the use of PBI-TB in a single adsorption stage alone needs unpractical amounts of this adsorber to reach the TTC. Still, the hybrid process is capable of providing a solution with less than 10% API loss using GMT-oNF-2 membrane and 76 g/L of PBI-TB. Whether the mitigation on API losses compensates the costs on additional stages required by the hybrid process will be the second case to be assessed on the economic analysis.

### 4.3. Economic and Environmental Analysis

#### 4.3.1. Process and Economic Model

An economic and environmental analyses were performed for the Meta purification processes comparing adsorption, OSN and the hybrid processes for two of the case studies considered:

(i) Removal of DMAP from Meta solutions using different single stage processes-adsorption using PBI-TA or OSN—Is compared by evaluating the balance between process costs and revenue losses due to API losses balance.
(ii) Removal of MPTS from Meta using only OSN or hybrid processes are compared to assess whether the recovery of the API on the hybrid process compensates the costs with additional stages.

The economic and environmental analyses were performed at a scale of 1 m^3^ implying a feed volume (V_F_) scale-up factor of 1000 times for all processes considered. API and GTI concentrations of the feed solutions were maintained at values of 10 g/L of API and 1000 mg/L of GTI. For the case of OSN, the selected membrane presents rejections of 90% for Meta and 10% for MPTS for Meta-MPTS solutions, and rejections corresponding to 96% for Meta and 40% for DMAP for Meta-DMAP solutions. Note that, such parameters were obtained at laboratory scale on dead-end mode, but scale-up using cross flow spiral wound modules should lead to reduction of concentration polarization and thus on apparent membrane rejections observed. For the adsorption step, the performance of PBI-TA as adsorber was selected for the single stage process for removal of DMAP (Langmuir isotherm with Q_max_ = 8.50 × 10^3^ gMeta/gPBI-TA and k_L_ = 2.1 L/g for Meta, and Q_max_ = 100 mgDMAP/gPBI-TA and k_L_ = 0.0081 L/g for DMAP). PBI-TB was selected as adsorber for the hybrid process (Freundlich isotherm with n = 2 and k_F_ = 0.0078 gAPI^1−1/n^/(gAdsorber.L^1/n^) for Meta and k_F_ = 0.1857 mgGTI^1−1/n^/(gAdsorber.L^1/n^) for MPTS). Adsorber isotherms were assumed to be volume independent, thus a factor of 1000 was equally used for the adsorption and hybrid processes, maintaining adsorber to solvent ratio at optimized values of 24.8 g/L PBI-TA for DMAP removal and 76 g/L PBI-TB on the hybrid process. For the hybrid process a 0.3 R_VRec/VF_ was used. The diavolumes were optimized to reach the 7.5 mgGTI/API at 3.2 diavolumes to remove MPTS using the hybrid process and 4.7 diavolumes to remove DMAP by OSN alone. Again, OSN transmembrane flux was also assumed to be scale independent and membrane areas were calculated accordingly, assuming the use of spiral wound membrane modules. Annual API purification was established at 450 kg of API, resulting from 90 batches taking place each year. Solvent recycling by distillation was assumed on all cases, with a solvent recovery efficiency of 95%.

Process flow diagrams designed for the three processes are represented in Figures S2–S4 for adsorption, OSN and hybrid process. Each process was modelled using SuperPro Design^TM^ and Microsoft Excel. The cost analysis includes the costs associated with (i) capital costs (i.e., equipment, equipment installation), (ii) maintenance, (iii) labour costs, (iv) selective agents (i.e., membrane and adsorbent), (v) solvents, and (vi) energy and utilities. A 10-year period was considered for the economic analysis and depreciations. The details on the model costs are provided in supplementary data (Table S8 to Table S10).

**Figure 7:**
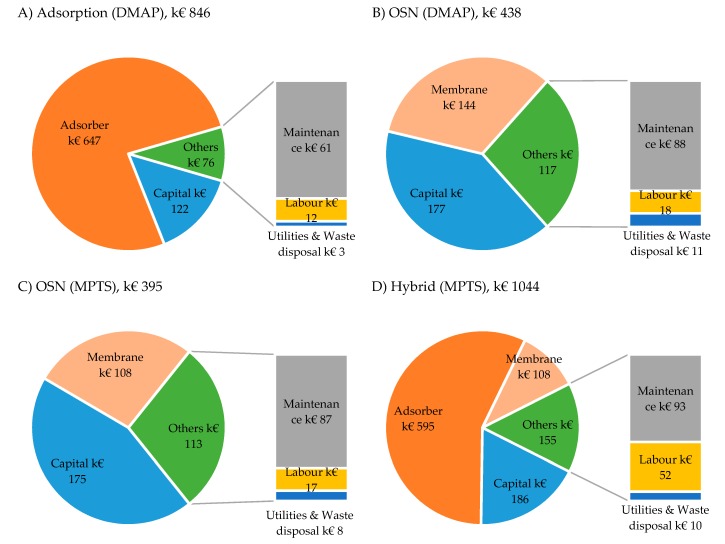
Annual cost distribution featuring the most significant contributions for the processes for DMAP removal by adsorption (**A**) and OSN (**B**), and the processes for MPTS removal by OSN (**C**) and hybrid process (**D**).

Total annual cost and corresponding cost distributions for each process are represented in Figure 7. The hybrid process has the highest capital costs and maintenance, since the main equipment includes both the filtration equipment present in the OSN process and the chromatography equipment present in the adsorption process. Different full time equivalent (FTE) and respective labour costs were computed for the several processes according with process operation times and complexity (Table S9).

The membrane cost at a value of 144 k€/year (Figure 7B) and 108 k€/year (Figure 7C) is the main consumable cost for OSN processes and the second highest for the hybrid process (Figure 7D). The cost of membrane is the same for the hybrid process and OSN for MPTS removal, as the same models were used but, with different operation times. The adsorber is the main cost for the adsorption and the hybrid processes at values of 647 and 595 k€/year, respectively. Still, this cost can be significantly decreased for cases where adsorber regeneration can be considered. On this particular analysis, it is assumed the use of fresh adsorber on each chromatographic column operation, with spent adsorber being discarded after this purification step finishes. The costs with adsorber are lower for the hybrid process than for the adsorption process alone, as the later uses higher amounts of adsorber.

**Figure 8:**
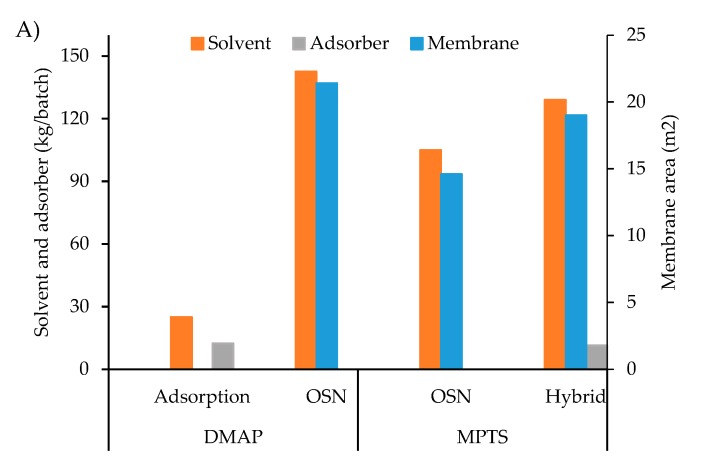

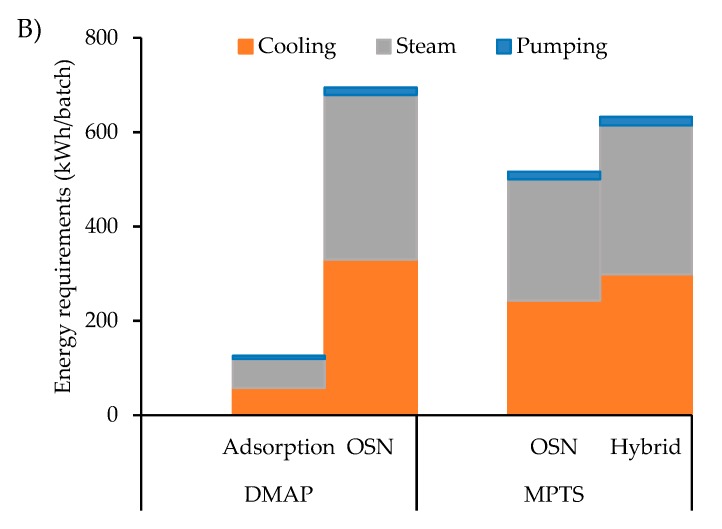
Comparison of (**A**) solvent and selective agent (adsorber or membrane), and (**B**) energy requirements per batch in terms of steam, cooling and pumping, for adsorption, OSN and hybrid processes.

The amount of solvent needed for MPTS removal is higher for the hybrid process than OSN (Figure 8A), as both processes require 3.2 diavolumes of DCM for the diafiltration unit operation, but the OSN stage in the hybrid process is fed with a higher volume, resulting of the combination of the API post reaction stream (V_F_) and the recirculation stream (ratio V_Rec_/V_F_ = 0.3) (Table S6). A lower amount of solvent is required on the adsorption process. For the removal of DMAP, solvent requirements are clearly lower for adsorption than OSN (Figure 8A), as the latter needs to process 4.7 diavolumes of post reaction stream (4.7 V_F_), while adsorption only processes V_F_. Note that for all the processes is assumed solvent recycling by distillation with 5% solvent make-up.

Calculations on energy requirements per batch (Figure 8B) result on around 4 times higher needs in steam for OSN and the hybrid processes than for the adsorption process, which is expected since most steam is used for solvent recycling by distillation. The same trend is followed by the cooling requirements. Energy requirements for pumping are due to input pressure to the filtration process and fluid transportation. Therefore, for DMAP removal processes, energy costs were higher for OSN than adsorption, due to higher solvent volumes being processed which lead to an increase in utilities’ cost coming from steam and cooling. The hybrid process also shows higher energy costs than OSN for MPTS removal, again due to higher solvent amount accounting for the recycle stream. Waste disposal costs were higher for the hybrid process than OSN, due to the extra expense of disposing of used adsorber.

In terms of total annual costs, the adsorption process (846 k€/year) is more expensive than OSN (438 k€/year) for removal of DMAP, while the hybrid process is costlier than OSN for MPTS removal, at 1044 k€/year and 395 k€/year, respectively (Figure 7). This difference is allocated to the cost of replacing the adsorber every new batch for the adsorption and hybrid processes, when compared to membrane replacement every 20 batches for the OSN processes. Additionally, capital investment is higher for the hybrid process than OSN, as previously discussed, due to the increased investment in equipment.

**Figure 9:**
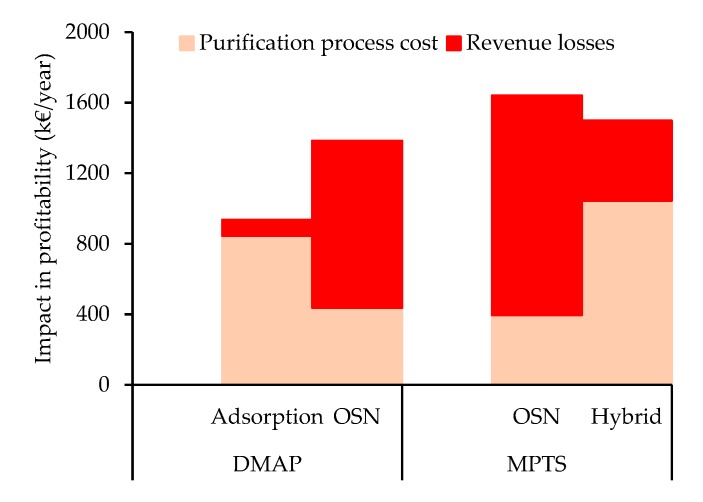
Comparison on percentage of revenue loss in terms of cost of purification treatment and cost of API loss in each purification process (adsorption, OSN and hybrid).

A simplified cost benefit analysis takes into account not only the cost for API purification treatment in each of the processes, but also the revenue losses due to API losses assuming an API value of 10 €/g (Figure 9).

**Figure 10:**
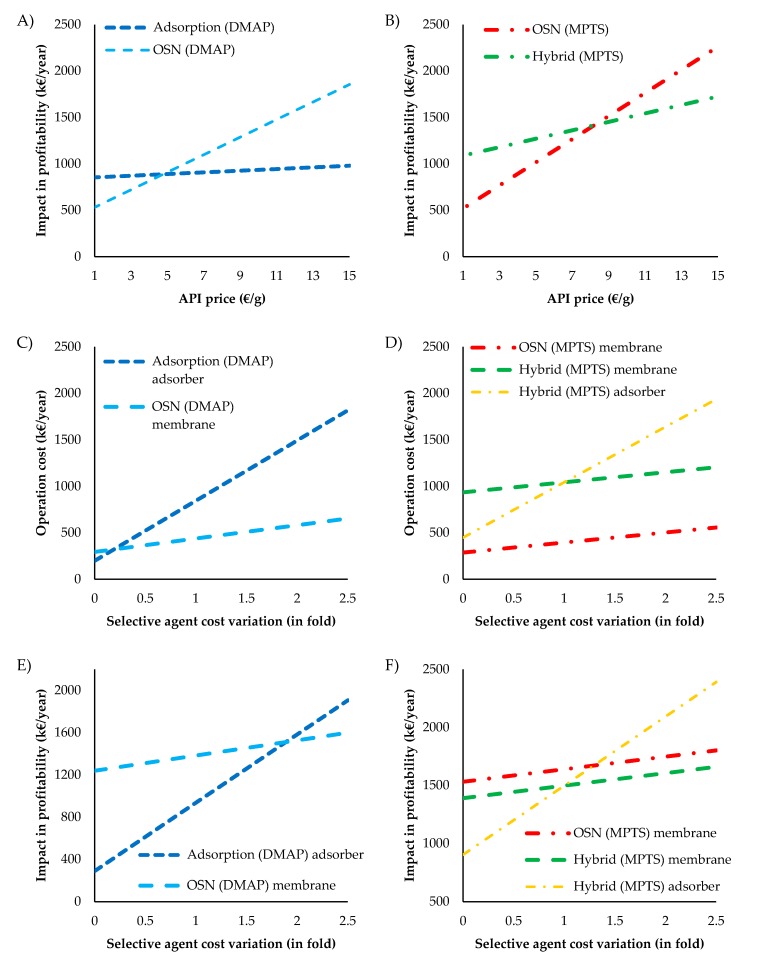
Sensitivity analysis of the four processes – adsorption and OSN for DMAP removal, OSN and hybrid for MPTS removal –, using variation of API price in terms of annual impact in profitability for DMAP removal (**A**) and MPTS removal (**B**), variation of selective agent (membrane or adsorber) cost reflected in annual cost of operation in processes for DMAP removal (**C**) and processes for MPTS removal (**D**), and annual impact in profitability for DMAP removal (**E**) and MPTS removal (**F**).

For removal of DMAP from a Meta solution, the total impact on profitability of the OSN process is higher than for the adsorption process. This result is driven from higher API losses for the OSN process, as actually the adsorption annual cost is actually higher than the OSN process (Figure 9). Note that, this result is dependent on API price and adsorption cost. The sensibility analysis to these parameters show that for APIs with a price below 4 €/g, the impact on profitability is lower using OSN than when adsorption is used (Figure 10A).

For the removal of MPTS from a Meta solution, the use of the more complex and costly hybrid process has a slightly lower impact on profitability than using the OSN process alone for a 10 €/g API. Indeed, the costs of implementing the hybrid process are compensated by the mitigation of API losses, for API worth more than 8 €/g. Moreover, API price increases has a significant higher impact on the OSN process than the hybrid process, as seen by the slope in the sensibility analysis (Figure 10B).

Additional sensibility analyses were made for the impact of the main consumable costs, up to 2.5-fold increases in membrane and adsorber unit costs on annual operation costs (Figure 10C,D) and, for a 10€/g API, profitability impact (Figure 10E,F).

Adsorber cost variation only has impact on the adsorption and hybrid processes, while membrane cost variations are relevant for both OSN processes and the hybrid process. For removal of DMAP from Meta (Figure 10C), an increase in adsorber cost will lead to a more significant increase in operation cost for the adsorption process, as this process presents a steeper slope than the membrane cost increase for OSN. It is interesting to note that operation cost of the adsorption process is below OSN only for cheaper adsorbers (below a quarter of the price of the present case study) (Figure 10C). However, the higher API losses associated to the OSN process, for a 10 €/g API, carry on to have a major impact on the total impact on profitability, implying that the adsorption cost would need to duplicate for compensating the use of OSN over the use of adsorption process to remove DMAP from a Meta solution (Figure 10E).

For the case of MPTS removal from a Meta solution, the impact of profitability (Figure 10F) considering API losses, for a 10 €/g API, and cost of operation (Figure 10C) of the hybrid or the OSN processes are not affected significantly by membrane price variation. However, the cost of adsorber has a clear impact on the yearly costs of the hybrid process. Actually, an increase of adsorber in 1.25 folds would turn the hybrid process too expensive to compete with the OSN process. On the other hand, if adsorber costs decreases, for example through adsorber regeneration, the hybrid process becomes an increasingly interesting option for removal of MPTS.

#### 4.3.2. Environmental Analysis

**Figure 11:**
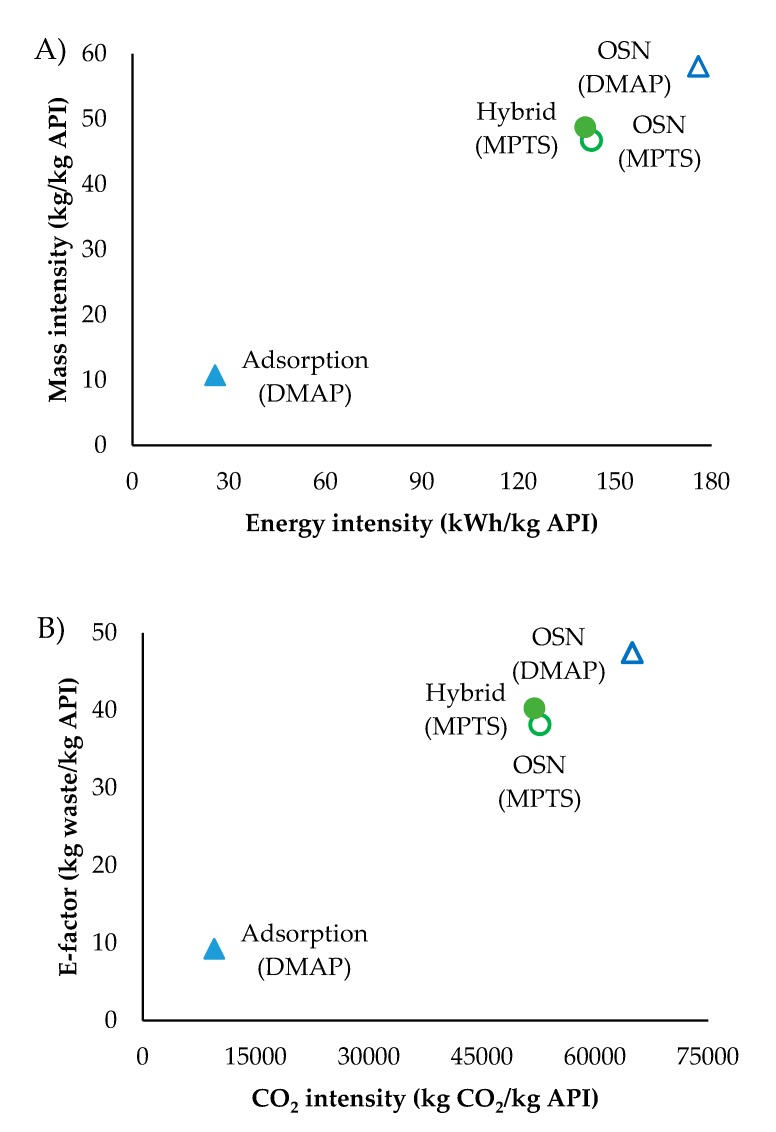
(**A**) Mass intensity and energy intensity metrics. (**B**) Environmental (E) factor and CO_2_ intensity metrics. Circles are used to compare the two processes for MPTS removal and triangles the two processes for DMAP removal.

An environmental analysis is here performed using selected green metrics to provide additional insights on process impacts. The green metrics selected include the environmental factor (E-factor), mass intensity, energy intensity and CO_2_ intensity. Mass intensity was calculated considering the mass of solvent used per kg of purified API [31]. The approach for energy intensity was similar, considering the energy requirements in steam, cooling and pumping per kg of API produced. These two green metrics were plotted in Figure 11A. The E-factor was determined by taking into account solid and liquid waste generated in each process per kg of API recovered [32]. The metric hereby called CO_2_ intensity was obtained by adding all sources of CO_2_ generated by the process (e.g., solvent waste that is not recycled and goes into incineration, CO_2_ associated with generation of electricity, steam and cooling used in the process), divided by the mass of purified API [11]. The green metrics, E-factor, and CO_2_ intensity are represented in Figure 11B.

OSN has a higher impact in terms of mass and energy than the adsorption process for DMAP. These observations were expected as higher volumes of solvent are processed, and energy is required to recycle it, in the diafiltration than in the adsorption process. Consistently, a lower E-factor and CO_2_ intensity was calculated for the adsorption process than for OSN processes for DMAP removal. Note that, although high, the OSN E-factor (47 kg waste/kg API) is still within the range of more than 100 kg waste/kg API [33] usually observed for pharmaceutical industry. The higher API losses on the OSN process than in the adsorption process also contributes to lower calculated metric values for the later process.

While the metrics here reported point out for a higher environment performance of DMAP removal by adsorption than for OSN, one needs to consider the scope of the metrics used before making a final conclusion. Namely, the E-factor and mass intensity takes into account both amount of adsorber and solvent used and disposed. However, the disposal of these materials has different environmental impacts, with controlled solvent incineration and heat recovery being facilitated, when compared to disposal of the adsorber. Moreover, the environmental impact of solvent, membrane and adsorber production is also not taken into account.

When comparing the processes for MPTS removal, the metrics calculated for OSN and the hybrid process are very similar. Although the energy and solvent requirements, are higher for the hybrid process than for the OSN process, lower API losses are observed on the former process, implying that, for the hybrid process, larger amount of mass and energy requirements are actually divided by a higher amount of API recovered, which is translated into similar green metrics for both processes analysed for MPTS removal from Meta solutions. The same trend is observed for CO_2_ and waste production metrics, which are similar for the hybrid process and OSN for MPTS removal. The use of higher amounts of solvent and the introduction of the adsorption step, results on higher generation of waste for the hybrid process than for the OSN process. However, similar E-factor for the OSN and hybrid processes results from the higher amount of API recovered by the later process. Again, according to Sheldon [33], E-factors around 40 kg waste/kg API are within the acceptable range for pharmaceutical industry [33]. The same happens with the CO_2_ generated.

## 5. Conclusions

This study presents a mathematical approach to support the decision on removing GTI from an API rich stream using an adsorption, OSN in diafiltration mode, or a hybrid process based on membrane rejections for API and GTI and isotherm adsorption constants. An important feature of the approach taken is that, these parameters have to be experimentally obtained for each system targeted. Importantly, some of such parameters may change with scale-up, as for example the values for apparent membrane rejections obtained with dead-end filtration should be different of the ones for crossflow spiral-wound membrane modules, due to reduction of concentration polarization in the crossflow mode. Therefore, it is recommended the use of data collected on the appropriate settings.

Once appropriate input data is collected, the framework here suggested allows optimization to meet a specific target ratio of impurity to product and calculate the losses on product. The value for the target ratio is dependent of industry standards. In the particular case tackled by the current study, the removal of a GTI from an API, the concept of TTC and the API recommended dosage for a specific application, was considered. However, such ratio needs to be defined for each specific purification targeted. Such optimization is made by calculation of the appropriate diavolumes or adsorber amounts for the OSN or adsorption process respectively or diavolumes, adsorber amounts and recirculation to feed ratio. However, such optimization also depends on the initial product and impurity concentration, which need to be imputed on the model for each decision-making process.

In this study, a target value of 7.5 mgGTI/gAPI was calculated to reach the TTC imposed by regulatory agencies and an initial concentration for 10 g/L of API and 1000 mg/L of GTI were assumed. The combination of membrane rejections for API and GTI or isotherm parameters leading to acceptable API losses were identified. The use of a hybrid process was suggested for the cases where OSN or adsorption would be ineffective or lead to non-acceptable API losses. The hybrid process here proposed includes an OSN stage, operated in diafiltration to remove GTI from an API stream, a distillation stage to concentrate the permeate and an adsorption stage to upgrade the bottom fraction of the distillation to a GTI/API ratio suitable to be back fed to the OSN stage. In this work, case studies are identified where OSN or adsorption processes would lead to 23–28% API losses for OSN or adsorption processes, but the use of the hybrid process can lead to 3.0 to 6.4% API losses using the same OSN membrane and adsorber. The model is used to select a recirculation to feed stream volume of 0.3 and adsorber amount of 20 g/L.

An experimental section was performed for case studies using two API, (Meta and Beta), two GTIs (DMAP and MTPS), two adsorbers (PBI-TA and PBI-TB) and one OSN membrane (GMT-oNF-2). The results obtained allowed to select as interesting cases for the economic and environmental analysis the comparison of using adsorption on PBI-TA vs OSN for removal of DMAP from a Meta solution, and OSN vs hybrid process for removal of MPTS from a Meta solution. The model was applied to calculate GTI removal and API losses, using membrane rejections and isotherm parameters obtained experimentally, to select the optimum diavolumes for OSN operation, amount of adsorber and recirculation to feed volume ratio. The economic analysis highlights trade-offs between process cost and revenue losses due to API losses and identifies API prices thresholds, important on decision making of which decontamination process to use. For example, while removal of DMAP using adsorption over OSN is recommended for API valued at more than 4 €/g, the use of the hybrid process is recommended over the OSN process to remove MPTS for API worth more than 8 €/g. The main consumable costs are associated with membrane and adsorber, with variations on costs of adsorber having an important effect on decision making. For example, for a 10 €/g API, DMAP removal by OSN, rather than adsorption, may become a preferable choice if adsorber costs increase 2-fold. For the same API value, MPTS removal by the hybrid process, rather than OSN, is a selection that makes sense as long as the adsorber does not increase more than 1.25-fold. The environmental analysis using selected green metrics is dominated by solvent use and respective recycling. Therefore, such metrics support the use of DMAP removal by adsorption and show similar environment performances for the hybrid and OSN process. Still, such metrics do not account for the different environmental impact of disposing solvents and adsorbers nor the environmental footprint on producing adsorber, membranes or solvents used on the suggested process.

## Figures and Tables

**Table 1:** API loss in diafiltration mode for different combinations of membrane rejections for API and GTI. Values for API losses below 10%, between 10% and 30% and above 30% in cells in green, yellow and red, respectively. Darker colour cells indicate the need for diavolumes above 5 to reach TTC.

		API Loss (%)						
		API Rejection						
		80%	85%	90%	95%	97.5%	99%	99.99%
**GTI rejection**	0%	47.7	36.7	25.0	12.7	6.4	2.6	0.0
	10%	52.3	40.4	**27.7**	14.1	7.1	2.9	0.0
	20%	57.8	45.0	30.9	15.9	8.0	3.2	0.0
	30%	64.5	50.7	35.1	18.1	9.1	3.7	0.0
	40%	72.6	57.8	40.4	21.0	10.7	4.3	0.0
	50%	82.2	67.0	47.7	25.0	12.7	5.1	0.0
	60%	92.5	78.9	57.8	30.9	15.9	6.4	0.0
	70%	99.4	92.5	72.6	40.4	21.0	8.5	0.0

**Table 2:** API loss related to each Langmuir adsorber. Values for API losses below 10%, between 10% and 30% and above 30% in cells in green, yellow and red, respectively. Darker colour cells indicate the need for adsorber loads higher than 15 %m/v to meet TTC value. n.d. represents combinations to which is impossible to reach TTC, regardless of the amount of adsorber used or no data, as no solution is found with values in ${\mathbb{R}}_{+}^{*}$. The adsorber used in the hybrid process is indicated in blue.

				API Loss (%)															
				Low <------------------------------------------------------------------------ API Adsorption Capacity ---------------------------------------------------------------------->High															
				Q_max_ = 0.0085 gAPI/gAdsorber				Q_max_ = 0.085 gAPI/gAdsorber				Q_max_ = 0.85 gAPI/gAdsorber				Q_max_ = 8.5 gAPI/gAdsorber			
	(mgGTI/gAdsorber)			A1	A2	A3	A4	B1	B2	B3	B4	C1	C2	C3	C4	D1	D2	D3	D4
			k_L, API_ (L/gAPI)	0.002 L/gAPI	0.021 L/gAPI	0.21 L/gAPI	2.1 L/gAPI	0.002 L/gAPI	0.021 L/gAPI	0.21 L/gAPI	2.1 L/gAPI	0.002 L/gAPI	0.021 L/gAPI	0.21 L/gAPI	2.1 L/gAPI	0.002 L/gAPI	0.021 L/gAPI	0.21 L/gAPI	2.1 L/gAPI
		k_L, GTI_ (L/mgGTI)																	
High <------GTI Adsorption capacity----------> Low	Q_max_ = 1	aI	0.0081	4.23	34.34	n.d.	n.d.	38.06	n.d.	n.d.	n.d.	n.d.	n.d.	n.d.	n.d.	n.d.	n.d.	n.d.	n.d.
		aII	0.081	1.86	14.45	54.60	n.d.	16.45	n.d.	n.d.	n.d.	n.d.	n.d.	n.d.	n.d.	n.d.	n.d.	n.d.	n.d.
		aIII	0.81	1.62	12.55	44.99	74.74	14.33	n.d.	n.d.	n.d.	n.d.	n.d.	n.d.	n.d.	n.d.	n.d.	n.d.	n.d.
		aIV	8.1	1.60	12.37	44.10	71.64	14.12	n.d.	n.d.	n.d.	n.d.	n.d.	n.d.	n.d.	n.d.	n.d.	n.d.	n.d.
	Q_max_ = 10	bI	0.0081	0.43	3.59	14.98	23.85	4.23	34.34	n.d.	n.d.	38.06	n.d.	n.d.	n.d.	n.d.	n.d.	n.d.	n.d.
		bII	0.081	0.19	1.57	6.16	8.89	1.86	14.45	54.60	n.d.	16.45	n.d.	n.d.	n.d.	n.d.	n.d.	n.d.	n.d.
		bIII	0.81	0.16	1.37	5.34	7.66	1.62	12.55	44.99	74.74	14.33	n.d.	n.d.	n.d.	n.d.	n.d.	n.d.	n.d.
		bIV	8.1	0.16	1.35	5.26	7.54	1.60	12.37	44.10	71.64	14.12	n.d.	n.d.	n.d.	n.d.	n.d.	n.d.	n.d.
	Q_max_ = 100	cI	0.0081	0.04	0.36	1.42	2.01	0.43	3.59	14.98	**23.85**	4.23	34.34	n.d.	n.d.	38.06	n.d.	n.d.	n.d.
		cII	0.081	0.02	0.16	0.62	0.88	0.19	1.57	6.16	8.89	1.86	14.45	54.60	n.d.	16.45	n.d.	n.d.	n.d.
		cIII	0.81	0.02	0.14	0.54	0.76	0.16	1.37	5.34	7.66	1.62	12.55	44.99	74.74	14.33	n.d.	n.d.	n.d.
		cIV	8.1	0.02	0.14	0.53	0.75	0.16	1.35	5.26	7.54	1.60	12.37	44.10	71.64	14.12	n.d.	n.d.	n.d.
	Q_max_ = 10,000	dI	0.0081	0.01	0.04	0.14	0.20	0.04	0.36	1.42	2.01	0.43	3.59	14.98	23.85	4.23	34.34	n.d.	n.d.
		dII	0.081	0.01	0.02	0.06	0.09	0.02	0.16	0.62	0.88	0.19	1.57	6.16	8.89	1.86	14.45	54.60	n.d.
		dIII	0.81	0.01	0.01	0.05	0.08	0.02	0.14	0.54	0.76	0.16	1.37	5.34	7.66	1.62	12.55	44.99	74.74
		dIV	8.1	0.01	0.01	0.05	0.08	0.02	0.14	0.53	0.75	0.16	1.35	5.26	7.54	1.60	12.37	44.10	71.64

**Table 3:** API loss related to Freundlich model with n = 1, 2 and 3. Values for API losses below 10%, between 10% and 30% and above 30% in cells in green, yellow and red, respectively. Darker colour cells indicate the need for adsorber loads higher than 15 %m/v to meet TTC value. n.d. represents combinations to which is impossible to reach TTC, regardless of the amount of adsorber used or no data, as no solution is found with values in ${\mathbb{R}}_{+}^{*}$. The adsorber used in the hybrid process is indicated in blue.

		n = 1						n = 2						n = 3					
	K_F,API_	0.001	0.01	0.05	0.10	0.25	0.50	0.001	0.01	0.05	0.10	0.25	0.50	0.001	0.01	0.05	0.10	0.25	0.50
K_F,GTI_																			
0.05		25.17	n.d	n.d	n.d	n.d	n.d	n.d	n.d	n.d	n.d	n.d	n.d	99.99	n.d	n.d	n.d	n.d	n.d
0.5		2.47	25.17	n.d	n.d	n.d	n.d	55.04	71.47	n.d	n.d	n.d	n.d	9.52	99.99	n.d	n.d	n.d	n.d
1		1.23	12.46	64.91	n.d	n.d	n.d	26.92	34.73	n.d	n.d	n.d	n.d	4.74	49.14	n.d	n.d	n.d	n.d
1.5		0.82	8.28	42.53	88.09	n.d	n.d	17.82	**22.94**	n.d	n.d	n.d	n.d	3.15	32.33	n.d	n.d	n.d	n.d
3		0.41	4.12	20.90	42.23	n.d	n.d	8.84	11.36	58.99	n.d	n.d	n.d	1.58	15.96	84.13	n.d	n.d	n.d
3.5		0.35	3.53	17.87	36.07	94.87	n.d	7.57	9.72	50.22	n.d	n.d	n.d	1.35	13.65	71.42	n.d	n.d	n.d
6		0.20	2.06	10.36	20.90	53.38	n.d	4.31	5.66	28.80	58.99	n.d	n.d	0.79	7.93	40.67	84.13	n.d	n.d
7.5		0.16	1.65	8.28	16.67	42.53	87.50	3.46	4.52	22.94	46.74	n.d	n.d	0.63	6.33	32.33	66.40	n.d	n.d
10		0.12	1.23	6.20	12.46	31.62	65.22	2.60	3.39	17.12	34.73	90.35	n.d	0.47	4.74	24.09	49.14	n.d	n.d
15		0.08	0.82	4.12	8.28	20.90	42.53	1,74	2.26	11.36	22.93	58.99	n.d	0.32	3.16	15.96	32.33	84.13	n.d
30		004	0.41	2.06	4.12	10.36	20.90	0.87	1.13	5.66	11.36	28.80	58.99	0.16	1.58	7.93	15.96	40.68	84.13

**Table 4:** Values for GTI/API in the permeate stream for different API/GTI rejections. Values for API losses below 10%, between 10% and 30% and above 30% in cells in green, yellow and red, respectively. Darker colour cells indicate the need for diavolumes above 5 to reach TTC.

		Permeate mgGTI/gAPI						
		API Rejection						
		80%	85%	90%	95%	97.5%	99%	99.99%
GTI rejection	0%	201.5	259.6	377.4 *	733.3	1447.0	3589.3	201.5
	10%	184.4	**236.3 ^+^**	**341.9**	662.0	1304.2	3232.2	184.4
	20%	**167.5 ^&^**	213.1	306.6	590.7	1161.4	2875.1	167.5
	30%	150.9	190.1	271.3	519.5	1018.7	2518.1	150.9
	40%	134.9	**167.5 ^&^**	**236.3 ^+^**	448.4	876.0	2161.0	134.9
	50%	120.0	145.5	201.5	377.4 *	733.3	1804.0	120.0
	60%	107.5	124.8	**167.5 ^&^**	306.5	590.7	1447.1	107.5
	70%	100.5	107.5	134.9	**236.3 ^+^**	448.4	1090.4	100.5

*,^+^,^&^—Similar API losses correspond to similar GTI/API ratios on the permeate stream.

**Table 5:** Comparison between predicted and experimental values obtained for the hybrid process.

Cycles	API Loss (%)		mgGTI/gAPI	
	Model	Experimental	Model	Experimental
1	27.66	24.73	7.5	7.25
2	14.96	16.03	7.5	7.08
3	11.38	9.76	7.5	6.62

## Supplementary Materials

The following are available online at https://www.mdpi.com/2077-0375/10/4/73/s1, Supporting information on: 1. Mathematical Section including variables and mathematical symbols as well as conditions imposed to model equations; 2. Chemical structure of PBI, PBI-TA and PBI-TB adsorbers; 3. Model results information, namely the diavolumes and amount of adsorber required to reach the target GTI/API ratio for different combinations of API and GTI rejections and isotherm parameters, respectively, as well as composition of adsorption inlet and outlet and OSN feed and permeate volumes for different ratios of recirculated to feed stream volumes, and; 4. Data and assumptions used for the economic and environmental analysis.
