# Supplementary material for: Comparison and Combination of Organic Solvent Nanofiltration and Adsorption Processes: A Mathematical Approach for Mitigation of Active Pharmaceutical Ingredient Losses during Genotoxin Removal
Source: Membranes (Basel). 2020 Apr 16;10(4):73. doi: 10.3390/membranes10040073 (PMC7231377; doi:10.3390/membranes10040073)
Supplement: Supplementary file 1 [file membranes-10-00073-s001.pdf]

Supporting Information

Comparison and Combination of Organic Solvent Nanofiltration and Adsorption Processes: A Mathematical Approach for Mitigation of Active Pharmaceutical Ingredient Losses during Genotoxin Removal

Flávio Ferreira, Leonor Resina, Teresa Esteves and Frederico Castelo Ferreira *

iBB—Institute for Bioengineering and Biosciences, Department of Bioengineering, Instituto Superior Técnico—Universidade de Lisboa, Avenida Rovisco Pais 1, 1049-001 Lisboa, Portugal; flavio.ferreira@tecnico.ulisboa.pt (F.F.); m.leonor.resina@tecnico.ulisboa.pt (L.R.); teresa.esteves@tecnico.ulisboa.pt (T.E.)

* Correspondence: frederico.ferreira@tecnico.ulisboa.pt; Tel.: +351-218419598

Contents:

1. Mathematical Section
   - 1.1 Variables and mathematical symbols
   - 1.2 Conditions imposed to model equations
2. Chemical structure of PBI, PBI-TA and PBI-TB
3. Model results
   - 3.1 Diavolumes required to reach the target GTI/API for different combinations of API and GTI rejections
   - 3.2 Adsorber amount required to reach the target GTI/API for different combinations of API and GTI isotherm parameters
   - 3.3 Effect of ratio VF_{Rec}/VF in composition of adsorption inlet and outlet and OSN feed and permeate volumes
4. Economic and environmental analysis
   - 4.1 Process flowsheets
   - 4.2 Economic analysis assumptions and inputs - additional details
   - 4.3 Equipment cost for adsorption, OSN and hybrid processes
   - 4.4 Full time equivalent (FTE) for each process and corresponding labour costs
   - 4.5 Membrane area requirements and corresponding replacement cost

1. Mathematical Section

1.1. Variables and mathematical symbols

$\mathbb{R}_+^*$ – positive real number not null

$\in$ – Belongs to

e – Euler's number (approximately 2.71828)

C_e – concentration of equilibrium at adsorption

C_F – concentration of feed

C_R – concentration of retentate

D – diavolume

F – Feed stream

F′ – feed for hybrid process cycles (initial feed stream + recycle stream)

MaxC – Maximum contamination

P – Permeate stream

P′ – Permeate after distillation

P′ – permeate after volume reduction

R – retentate stream

Rec – recycle stream

Rej – rejection

V – volume

Vadd – added volume

Subscribed letters

x – index for generic chemical specie (could be replaced for API or GTI)

i – index for current system (could be replaced for diavolume number to OSN or the adsorber used in adsorption)

j – index for cycle number

1.2. Conditions Imposed to Model Equations

• **For OSN:** Equation 5 allows the calculation of diavolumes to reach a given target value of decontamination in terms of mgGTI/gAPI from solutes rejections and initial concentrations. Here, it is specified how equation 5 is obtained from combination of equations 1 and 4. Equation 4 was rearranged to be a function of C_R applied for GTI and API, resulting on:

$C_{Rej,GTI} = C_{F,GTI}.e^{[-D_i(1-Rej_{GTI})]}$ and $C_{R,API} = C_{F,API}.e^{[-D_i(1-Rej_{API})]}$

Applying both equations to equation 1 will generate $MaxC = \frac{C_{F,GTI}.e^{[-D_i(1-Rej_{GTI})]}}{C_{F,API}.e^{[-D_i(1-Rej_{API})]}}$ and finally, equation 6b appears after rearranging this equation as function of D_i.

• **For Langmuir:** For adsorptions following the Langmuir isotherm, the concentrations of solutes can be calculated by equation 11. However, concentrations should be real, zero or positive numbers, i.e. $C_{e,x,i} \in \mathbb{R}_+^*$. Therefore, the following conditions of existence were imposed to equation 11:

$$C_{e,x,i} = \frac{-V - m.Q_{max}.k_{L,x,i} + k_{L,x,i}.C_{in,x,i}.V}{2k_{L,x,i}.V} \pm \frac{\sqrt{V^2 + 2m.Q_{max}.k_{L,x,i}.V + 2C_{in,x,i}.k_{L,x,i}.V^2 - 2m.Q_{max}.C_{in,x,i}.k_{L,x,i}.V + k_{L,x,i}^2.C_{in,x,i}^2.V^2 + m^2.Q_{max}^2.k_{L,x}^2}}{2k_{L,x,i}.V}$$

The expression inside the square root needs to be positive to not result on imaginary numbers, i.e.:

$V^2 + 2m.Q_{max}.k_{L,x,i}.V + 2C_{in,x,i}.k_{L,x,i}.V^2 - 2m.Q_{max}.C_{in,x,i}.k_{L,x,i}.V + k_{L,x,i}^2.C_{in,x,i}^2.V^2 + m^2.Q_{max}^2.k_{L,x}^2 \geq 0$, to not have an imaginary number as answer.

The solution resulting from the **addition** of the two fractions on equation 1 need to be positive and therefore, when $\frac{-V-m.Q_{max}.k_{L,x,i}+k_{L,x,i}.C_{in,x,i}.V}{2k_{L,x,i}.V} < 0$:

$$\frac{\sqrt{V^2+2m.Q_{max}.k_{L,x,i}.V+2C_{in,x,i}.k_{L,x,i}.V^2-2m.Q_{max}.C_{in,x,i}.k_{L,x,i}.V+k_{L,x,i}^2.C_{in,x,i}^2.V^2+m^2.Q_{max}^2.k_{L,x}^2}}{2k_{L,x,i}.V} > \frac{-V-m.Q_{max}.k_{L,x,i}+k_{L,x,i}.C_{in,x,i}.V}{2k_{L,x,i}.V}$$

The solution resulting from the **subtraction** of the two fractions on equation 1 need to be positive and therefore, when:

a) $\frac{-V-m.Q_{max}.k_{L,x,i}+k_{L,x,i}.C_{in,x,i}.V}{2k_{L,x,i}.V} < 0$, the solution is not possible for that domain

b) $\frac{-V-m.Q_{max}.k_{L,x,i}+k_{L,x,i}.C_{in,x,i}.V}{2k_{L,x,i}.V} > 0$,

$$\frac{\sqrt{V^2+2m.Q_{max}.k_{L,x,i}.V+2C_{in,x,i}.k_{L,x,i}.V^2-2m.Q_{max}.C_{in,x,i}.k_{L,x,i}.V+k_{L,x,i}^2.C_{in,x,i}^2.V^2+m^2.Q_{max}^2.k_{L,x}^2}}{2k_{L,x,i}.V} <$$

$$\frac{-V-m.Q_{max}.k_{L,x,i}+k_{L,x,i}.C_{in,x,i}.V}{2k_{L,x,i}.V}$$

• **For Freundlich:** For adsorptions following the Freundlich isotherm, the concentrations of solutes can be calculated by equations 12-13. However, concentrations should be real, zero or positive numbers, i.e. $C_{e,x,i} \in \mathbb{R}_+^*$. Therefore, the following conditions of existence were imposed to the equations obtained for the models:

n=2: $$C_{e,x,i} = \frac{2C_{in,x,i}.V^2+m^2.k_{F,x,i}^2 \pm \sqrt{4C_{in,x,i}.m^2.k_{F,x,i}^2.V^2+m^4.k_{F,x,i}^4}}{2V^2}$$

All variables of the expression inside the square root are positive, it is not needed to impose a condition for existence to result on real numbers. Sill, to obtain a positive number, from the calculation resulting from the **subtraction** of the two fractions of the equation above, is required:

$$2C_{in,x,i}.V^2 + m^2.k_{F,x,i}^2 > \sqrt{4C_{in,x,i}.m^2.k_{F,x,i}^2.V^2 + m^4.k_{F,x,i}^4}$$

n=3:

$$C_{e,x,i} = \sqrt[3]{\frac{-C_{in,x,i}.m^3.k_{F,x,i}^3}{2V^3} + \sqrt{\frac{C_{in,x,i}^2.m^6.k_{F,x,i}^6}{4V^6} + \frac{m^9.k_{F,x,i}^9}{27V^9}}} + \sqrt[3]{\frac{-C_{in,x,i}.m^3.k_{F,x,i}^3}{2V^3} - \sqrt{\frac{C_{in,x,i}^2.m^6.k_{F,x,i}^6}{4V^6} + \frac{m^9.k_{F,x,i}^9}{27V^9}}} + C_{in,x,i}$$

When, the cubic root results in a negative number, to obtain a real positive number for $C_{e,x,i}$, is required that:

a) When the first cubic square results in negative value, then:

$$\sqrt[3]{\frac{-C_{in,x,i}.m^3.k_{F,x,i}^3}{2V^3} - \sqrt{\frac{C_{in,x,i}^2.m^6.k_{F,x,i}^6}{4V^6} + \frac{m^9.k_{F,x,i}^9}{27V^9}}} + C_{in,x,i} > \sqrt[3]{\frac{-C_{in,x,i}.m^3.k_{F,x,i}^3}{2V^3} + \sqrt{\frac{C_{in,x,i}^2.m^6.k_{F,x,i}^6}{4V^6} + \frac{m^9.k_{F,x,i}^9}{27V^9}}}$$

b) When the second cubic root results on negative values, then:

$$\sqrt[3]{\frac{-C_{in,x,i}.m^3.k_{F,x,i}^3}{2V^3} + \sqrt{\frac{C_{in,x,i}^2.m^6.k_{F,x,i}^6}{4V^6} + \frac{m^9.k_{F,x,i}^9}{27V^9}}} + C_{in,x,i} > \sqrt[3]{\frac{-C_{in,x,i}.m^3.k_{F,x,i}^3}{2V^3} - \sqrt{\frac{C_{in,x,i}^2.m^6.k_{F,x,i}^6}{4V^6} + \frac{m^9.k_{F,x,i}^9}{27V^9}}}$$

2. Chemical Structure of PBI, PBI-TA and PBI-TB

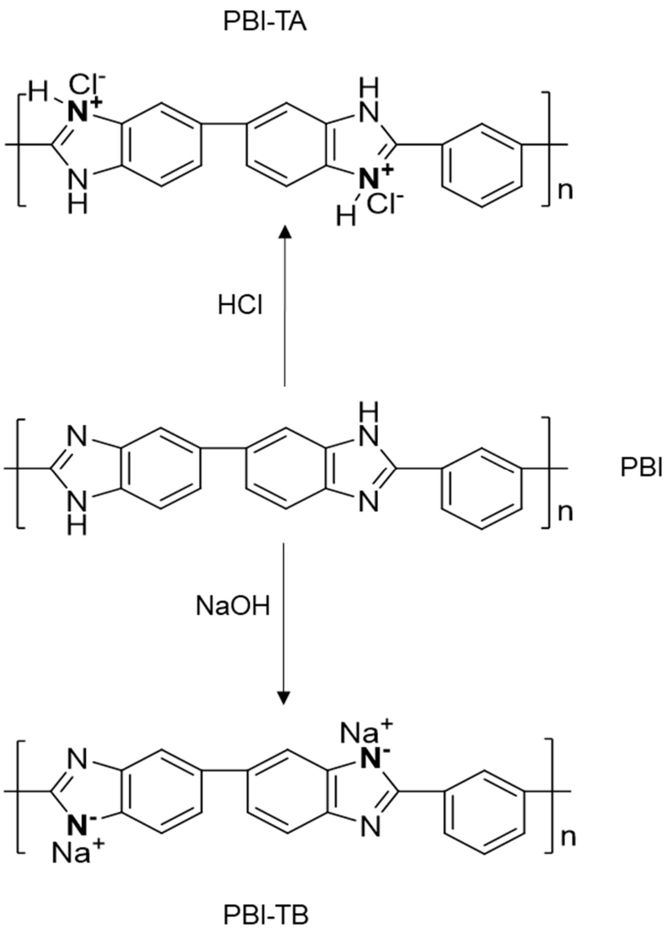

Figure S1. Chemical structure of PBI, PBI-TA and PBI-TB [1].

3. Model Results

3.1. Diavolumes Required to Reach the Target GTI/API for Different Combinations of API and GTI Rejections

Table 1. Diavolumes required for different combinations of API and GTI rejections. In grey is presented cases that need more than 5 diavolumes to reach the target value of 7.5 mgGTI/gAPI. In blue is highlighted the combination of membrane rejections for API and GTI used for calculations on the hybrid process.

		Diavolumes						
		API Rejection						
		80%	85%	90%	95%	97.5%	99%	99.99%
GTI rejection	0%	3.2	3.0	2.9	2.7	2.7	2.6	2.6
	10%	3.7	3.5	3.2	3.0	3.0	2.9	2.9
	20%	4.3	4.0	3.7	3.5	3.3	3.3	3.2
	30%	5.2	4.7	4.3	4.0	3.8	3.8	3.7
	40%	6.5	5.8	5.2	4.7	4.5	4.4	4.3
	50%	8.6	7.4	6.5	5.8	5.5	5.3	5.2
	60%	13.0	10.4	8.6	7.4	6.9	6.6	6.5
	70%	25.9	17.3	13.0	10.4	9.4	8.9	8.6

Table S2. Combination of membrane rejections for GTI and API using reported experimental values [2}. Membrane and solvent used were, respectively, GMT-oNF-2 and tetrahydrofuran.

	Number of combinations of membrane rejections for GTI and API							
	API Rejection							
	80%	85%	88.6% LA	90%	95% Suma	97.5% Pred; Beta	99% Irb; Halo; Meta	99.99% Roxi
**GTI rejection**								
0-2.5% (ACR; AA)			2		2	4	6	2
10% (DMCC)			1		1	2	3	1
15-20% (IPU; BE; TA)			3		3	6	9	3
20% (EtMS; DMS)			2		2	4	6	2
30%								
36.5% (MeTS)			1		1	2	3	1
40%								
50%								
56.5% (EtTS)			1		1	2	3	1
60%								
70%								

API: Lacosamide (LA), Sumatriptan (Suma), Irbesartan (Irb), Prednisolone (Pred), Betamethasone acetate (Beta), Halobetasole propionate (Halo), Mometasone furoate (Meta) and Roxithromycin (Roxi). GTI: methyl tosilate (MeTS), dimethylsulfate (DMS), acetamide (AA), thioacetamide (TA), dimethyl carbamoyl chloride (DMCC), 1,3-dicyclohexylurea (CHU), acrolein (ACR), 2-bromoethanol (BE), ethyl tosylate (EtTS), 1,3-diisopropylurea (IPU) and ethyl mesylate (EtMS).

3.2. Adsorber Amount Required to Reach the Target GTI/API for Different Combinations of API and GTI Isotherm Parameters

Table 3. Selected Langmuir's constants for API and GTI.

	Q_{max} (gAPI/gAdsorber; mgGTI/gAdsorber)		K_L (L/gAPI; L/mg GTI)	
	**Code**	**Value**	**Code**	**Value**
API	A	0.0085	1	0.0021
	B	0.085	2	0.021
	C	0.85	3	0.21
	D	8.5	4	2.1
GTI	A	1	I	0.0081
	B	10	II	0.081
	C	100	III	0.81
	D	1000	IV	8.1

Table S4. Required mass calculated for each combination of GTI and API Langmuir isotherms. In grey are values that need more than 15%m/v of adsorber to reach TTC and in dark grey values that are higher than 90 %m/v and thus, physically impossible solutions. In blue is highlighted the combination of isotherms for API and GTI used for calculations on the hybrid process. n.d. – no data, as no solution is found with values in $\mathbb{R}_{+}^{*}$.

High <------GTI Adsorption capacity------> Low (mgGTI/gAdsorber)			**Adsorber Mass (g/L)**															
			**Low <------ API Adsorption Capacity ------>High**															
			**Q_{max} = 0.0085 gAPI/gAdsorber**				**Q_{max} =0.085 gAPI/gAdsorber**				**Q_{max} =0.85 gAPI/gAdsorber**				**Q_{max} =8.5 gAPI/gAdsorber**			
			**A1**	**A2**	**A3**	**A4**	**B1**	**B2**	**B3**	**B4**	**C1**	**C2**	**C3**	**C4**	**D1**	**D2**	**D3**	**D4**
		$k_{L,\ GTI}$ (L/gGTI) \ $k_{L,\ API}$ (L/gAPI)	0.002 L/gAPI	0.021 L/gAPI	0.21 L/gAPI	2.1 L/gAPI	0.002 L/gAPI	0.021 L/gAPI	0.21 L/gAPI	2.1 L/gAPI	0.002 L/gAPI	0.021 L/gAPI	0.21 L/gAPI	2.1 L/gAPI	0.002 L/gAPI	0.021 L/gAPI	0.21 L/gAPI	2.1 L/gAPI
Q_{max} = 1	aI	0.0081	2523.49	3334.42	n.d.	n.d.	3487.85	n.d.	n.d.	n.d.	n.d.	n.d.	n.d.	n.d.	n.d.	n.d.	n.d.	n.d.
	aII	0.081	1081.77	1115.89	1316.20	n.d.	1122.00	n.d.	n.d.	n.d.	n.d.	n.d.	n.d.	n.d.	n.d.	n.d.	n.d.	n.d.
	aIII	0.81	941.71	952.01	987.43	1044.97	953.72	n.d.	n.d.	n.d.	n.d.	n.d.	n.d.	n.d.	n.d.	n.d.	n.d.	n.d.
	aIV	8.1	927.75	936.03	960.90	984.41	937.38	n.d.	n.d.	n.d.	n.d.	n.d.	n.d.	n.d.	n.d.	n.d.	n.d.	n.d.
Q_{max} = 10	bI	0.0081	245.50	251.17	274.88	298.10	252.35	333.44	n.d.	n.d.	348.78	n.d.	n.d.	n.d.	n.d.	n.d.	n.d.	n.d.
	bII	0.081	107.77	108.11	109.27	110.00.	108.18	111.59	131.62	n.d.	112.20	n.d.	n.d.	n.d.	n.d.	n.d.	n.d.	n.d.
	bIII	0.81	94.04	94.15	94.52	94.73	94.17	95.20	98.74	104.50	95.37	n.d.	n.d.	n.d.	n.d.	n.d.	n.d.	n.d.
	bIV	8.1	92.66	92.76	93.06	93.23	92.77	93.60	96.09	98.44	93.74	n.d.	n.d.	n.d.	n.d.	n.d.	n.d.	n.d.
Q_{max} = 100	cI	0.0081	24.48	24.54	24.72	24.83	24.55	25.12	27.49	**29.81**	25.23	33.34	n.d.	n.d.	34.88	n.d.	n.d.	n.d.
	cII	0.081	10.77	10.78	10.79	10.79	10.78	10.81	10.93	11.00	10.82	11.16	13.16	n.d.	11.22	n.d.	n.d.	n.d.
	cIII	0.81	9.40	9.40	9.41	9.41	9.40	9.41	9.45	9.47	9.42	9.52	9.87	10.45	9.54	n.d.	n.d.	n.d.
	cIV	8.1	9.27	9.27	9.27	9.27	9.27	9.28	9.31	9.32	9.28	9.36	9.61	9.84	9.37	n.d.	n.d.	n.d.
Q_{max} = 10000	dI	0.0081	2.45	2.45	2.45	2.45	2.45	2.45	2.47	2.48	2.46	2.51	2.75	2.98	2.52	3.33	n.d.	n.d.
	dII	0.081	1.08	1.08	1.08	1.08	1.08	1.08	1.08	1.08	1.08	1.08	1.09	1.10	1.08	1.12	1.32	n.d.
	dIII	0.81	0.94	0.94	0.94	0.94	0.94	0.94	0.94	0.94	0.94	0.94	0.95	0.95	0.94	0.95	0.99	1.04
	dIV	8.1	0.93	0.93	0.93	0.93	0.93	0.93	0.93	0.93	0.93	0.93	0.93	0.93	0.93	0.94	0.96	0.98

Table S5. Required mass calculated for each combination of GTI and API Freundlich isotherms. In grey are values that need more than 15%m/v of adsorber to reach TTC and in dark grey values that are higher than 90%m/v and thus, physically impossible solutions. In blue is highlighted the combination of isotherms for API and GTI used for calculations on the hybrid process. n.d. – no data, as no solution is found with values in $\mathbb{R}_{+}^{*}$.

Adsorber mass (g/L)																		
	n = 1						n = 2						n = 3					
K_{API} / K_{GTI}	0.001	0.01	0.05	0.10	0.25	0.50	0.001	0.01	0.05	0.10	0.25	0.50	0.001	0.01	0.05	0.10	0.25	0.50
0.05	336.4	n.d	n.d	n.d	n.d	n.d	n.d	n.d	n.d	n.d	n.d	n.d	3584685.7	n.d	n.d	n.d	n.d	n.d
0.5	25.3	33.6	n.d	n.d	n.d	n.d	332.8	423.1	n.d	n.d	n.d	n.d	457.1	233375.6	n.d	n.d	n.d	n.d
1	12.5	14.2	37.0	n.d	n.d	n.d	127,7	135.9	n.d	n.d	n.d	n.d	223.8	285.7	n.d	n.d	n.d	n.d
1.5	8.3	9.0	14.8	74.0	n.d	n.d	79.7	82.6	n.d	n.d	n.d	n.d	148.2	170.9	n.d	n.d	n.d	n.d
3	4.1	4.3	5.3	7.3	n.d	n.d	37.6	38.2	58.3	n.d	n.d	n.d	73.6	78.5	144.3	n.d	n.d	n.d
3.5	3.5	3.7	4.4	5.6	74.0	n.d	31.9	32.4	45.0	n.d	n.d	n.d	63.0	66.5	100.6	n.d	n.d	n.d
6	2.1	2.1	2.3	2.6	4.6	n.d	17.9	18.4	21.6	29.1	n.d	n.d	36.7	37.8	44.9	72.1	n.d	n.d
7.5	1.6	1.7	1.8	2.0	3.0	14.0	14.3	14.6	16.5	20.3	n.d	n.d	29.3	30.0	34.2	44.3	n.d	n.d
10	1.2	1.3	1.3	1.4	1.9	3.8	10.7	10.9	11.9	13.6	36.8	n.d	22.0	22.4	24.5	28.6	n.d	n.d
15	0.8	0.8	0.9	0.9	1.1	1.5	7.1	7.2	7.6	8.3	11.7	n.d	14.6	14.8	15.7	17.1	28.9	n.d
30	0.4	0.4	0.4	0.4	0.5	0.5	3.6	3.6	3.7	3.8	4.3	5.8	7.3	7.4	7.6	7.8	9	14.4

Notes:

- Mass values in Table S4 are calculated algebraically or iteratively for n=1 (Eq. 12) and iteratively for n= 2 and 3, using respectively Eqs. 13 and 14. For iterative calculations, the "solver" mathematical function from Excel, version 2013, was used to obtain the GTI and API concentrations in solution at equilibrium with the adsorber, i.e. $C_{e,API,i}$ and $C_{e,GTI,i}$, and from those values the mgGTI/gAPI ratio was calculated. That calculation is performed with given values for all the variables (initial concentrations of API and GTI, solution volumes) and parameters (isotherm constants for API and GTI) and an arbitrary first value for the mass of adsorber. Then, the calculation is repeated successively, changing the value of adsorber until a value is found for the mass of adsorber to which the GTI and API concentrations meet the target value of mgGTI/gAPI. Such value of adsorber is stored as final solution presented on this table and used to calculate API losses. The procedure is repeated 10 times with different initial values of adsorber to confirm that the final result is consistent.
- For isotherms corresponding to lower affinities of the GTI and the API to the adsorber, the model is still able to compute a mass of adsorber that would be able to meet the target value of mgGTI/gAPI. However, some of the values would go beyond mass that would fit physically on a solution, and therefore such values (at dark grey on Tables S3 and S4) should be discarded as possible results.

3.3 Effect of Ratio VF_{Rec}/VF in Composition of Adsorption Inlet and Outlet and OSN Feed and Permeate Volumes

Table S6. Effect of ratio VF_{Rec}/VF in adsorption in stream compositions for OSN inlet (cycle 1 and 10) and adsorption inlet and outlet (cycle 10) streams. Calculations assumed the use of 20 g/L of adsorber. The isotherms for GTI and API assumed were the ones corresponding to the Langmuir case B4cI model or the ones corresponding to Freundlich model with n=2, k_{FAPI}=0.01 $gAPI^{1-1/n}/(gAdsorber.L^{1/n})$ and k_{FGTI}=1.5 $mgGTI^{1-1/n}/(gAdsorber.L^{1/n})$. Two cases were considered for VF_{Rec}/VF = 0.3 (at red), one using 20 g/L and another using 40 g/L (marked with *) of adsorber.

		Adsorption Step Cycle 10							OSN inlet					
		Inlet			Outlet				API (g/L)		GTI (mg/L)		mgGTI/gAPI	
	VF_{Rec}/VF	API (g/L)	GTI (mg/L)	mgGTI/gAPI	API (g/L)	GTI (mg/L)	API loss (%)	mgGTI/gAPI	Cycle 1	Cycle 10	Cycle 1	Cycle 10	Cycle 1	Cycle 10
Langmuir	0.05	135.36	168.17	1242.39	133.67	166.17	2.82	1243.18	12.08	15.89	1.76	8.87	145.60	557.96
	0.10	65.29	75.20	1151.74	63.60	73.20	3.58	1150.90	11.45	14.87	1.59	7.56	138.82	508.55
	0.20	29.99	28.83	961.47	28.32	26.84	5.05	947.93	10.37	13.05	1.30	5.31	125.62	406.59
	0.30	17.90	13.61	760.30	16.25	11.63	6.37	715.79	9.45	11.44	1.07	3.45	113.70	301.79
	0.30*	11.05	3.54	320.02	7.85	0.43	9.62	54.77	9.09	9.50	0.84	0.87	92.34	91.38
	0.50	7.69	3.06	398.26	6.11	1.24	8.11	203.27	8.00	8.70	0.79	1.08	99.16	124.16
	0.75	4.42	1.45	327.58	2.96	0.20	10.98	68.79	6.69	6.98	0.64	0.66	95.20	94.34
	1.00	3.21	1.04	325.46	1.85	0.11	13.53	58.48	5.74	5.93	0.55	0.55	95.12	93.51
Freundlich	0.05	123.93	121.02	976.50	121.72	111.02	2.50	912.08	12.09	15.19	1.68	5.83	138.72	384.04
	0.10	58.84	50.71	861.92	57.32	44.39	2.66	774.42	11.51	14.20	1.54	4.68	133.90	329.40
	0.20	27.35	19.93	728.77	26.32	16.12	2.87	612.47	10.52	12.65	1.34	3.38	127.84	267.35
	0.30	17.27	11.18	647.22	16.46	8.42	3.04	511.82	9.68	11.44	1.20	2.63	123.73	230.26
	0.30*	13.70	5.72	417.45	12.30	2.64	4.26	931.45	9.56	10.53	1.03	1.37	107.84	130.63
	0.50	9.56	5.25	548.79	8.96	3.48	3.34	388.04	8.36	9.63	0.99	1.80	118.07	186.59
	0.75	5.94	2.85	480.41	5.47	1.64	3.68	299.54	7.14	8.05	0.81	1.26	113.34	157.08
	1.00	4.24	1.86	439.61	3.85	0.94	4.01	245.00	6.23	6.92	0.68	0.97	109.96	139.85

Table S7. Ratio between volume parameters on OSN outlet at cycle 10 and permeate volume, using 20 g/L of Langmuir B4cI model or Freundlich model with n=2, k_{FAPI}=0.01 gAPI$^{1-1/n}$/(gAdsorber.L$^{1/n}$) and k_{FGTI}=1.5 mgGTI$^{1-1/n}$/(gAdsorber.L$^{1/n}$). Two cases were considered for VF_{Rec}/VF = 0.3 (in red), one using 20 g/L and another using 40 g/L (marked with *) of adsorber. CF - Concentration factor.

	OSN outlet: Cycle 10					
	VF_{Rec}/VF	D = VP/VF'	VP/VF	$VP/VP_{OSN\ alone}$	VFRec/VP (%)	CF
Langmuir	0.05	5.29	5.55	1.71	1%	100.0
	0.10	5.17	5.69	1.76	2%	50.0
	0.20	4.9	5.88	1.82	3%	33.3
	0.30	4.53	5.89	1.82	5%	20.0
	0.30*	3.13	4.06	1.25	7%	14.3
	0.50	3.49	5.24	1.62	10%	10.0
	0.75	3.16	5.54	1.71	14%	7.1
	10	3.15	6.31	1.95	16%	6.3
Freundlich	0.05	4.92	5.17	1.60	1%	100.0
	0.10	4.73	5.2	1.61	2%	50.0
	0.20	4.47	5.36	1.66	4%	25.0
	0.30	4.28	5.56	1.72	5%	20.0
	0.30*	3.57	4.64	1.43	6%	16.7
	0.50	4.02	6.03	1.86	8%	12.5
	0.75	3.8	6.65	2.05	11%	9.1
	1.00	3.66	7.31	2.26	14%	7.1

4. Economic and Environmental Analysis

4.1. Process Flowsheets

Figures S2, S3 and S4 represent, respectively, the process diagrams for the 3 processes, Adsorption alone, OSN alone and the hybrid process. Storage 1/S1 represents an auxiliary storage tank in all processes, for the contaminated API stream coming from the upstream process. Storage 2/S2 works as an auxiliary storage tank for purified API in solution (S2) or an auxiliary storage tank for permeate after a (dia)nanofiltration step, containing low API and high GTI concentrations (S3 and S4). Common to all processes, Storage 3/S3 is an auxiliary storage tank for recycled solvent after distillation; Mixing/MX1 represents a valve that works as a mixing knot for recycled and make-up solvent streams; Distillation 1/D1 and Distillation 2/D2 represent distillation columns for solvent evaporation for recycling; and Condenser 1/C1 and Condenser 2/C2 are heat exchangers for condensation of the recycled solvent. In A and C, the equipment called Adsorption/A1 is a chromatographic column used for separation of GTI and API, letting most GTI be adsorbed and API to be eluted with the solvent. Diafiltration/DF1 operation units in B and C represent organic solvent (dia)nanofiltration equipment, including a tank for diavolume solvent. The Tray Drying/TD1 operation unit is a tray drying equipment used for the remaining solvent removal from purified API. For all processes, Pump 1/P1 to Pump 8/P8 are centrifugal pumps used for transport; and S-101 to S-127 designate process streams.

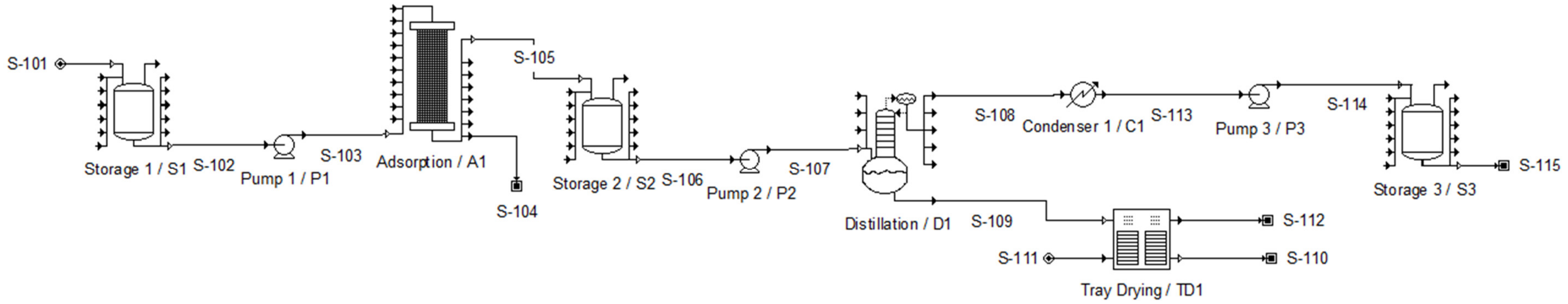

Figure S2. Process flow diagram for the adsorption process.

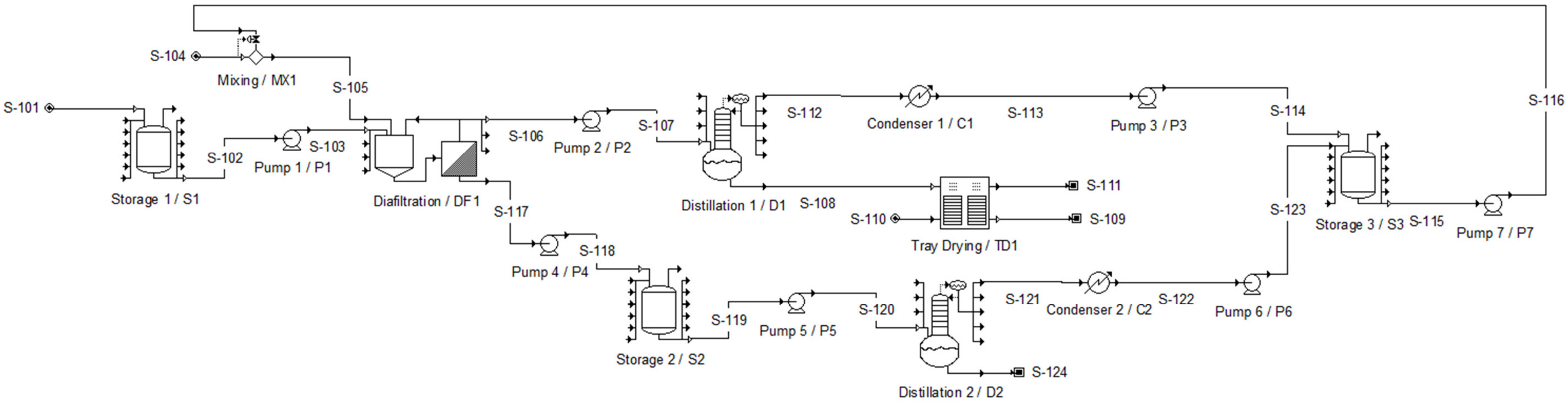

Figure S3. Process flow diagram for organic solvent (dia)nanofiltration process.

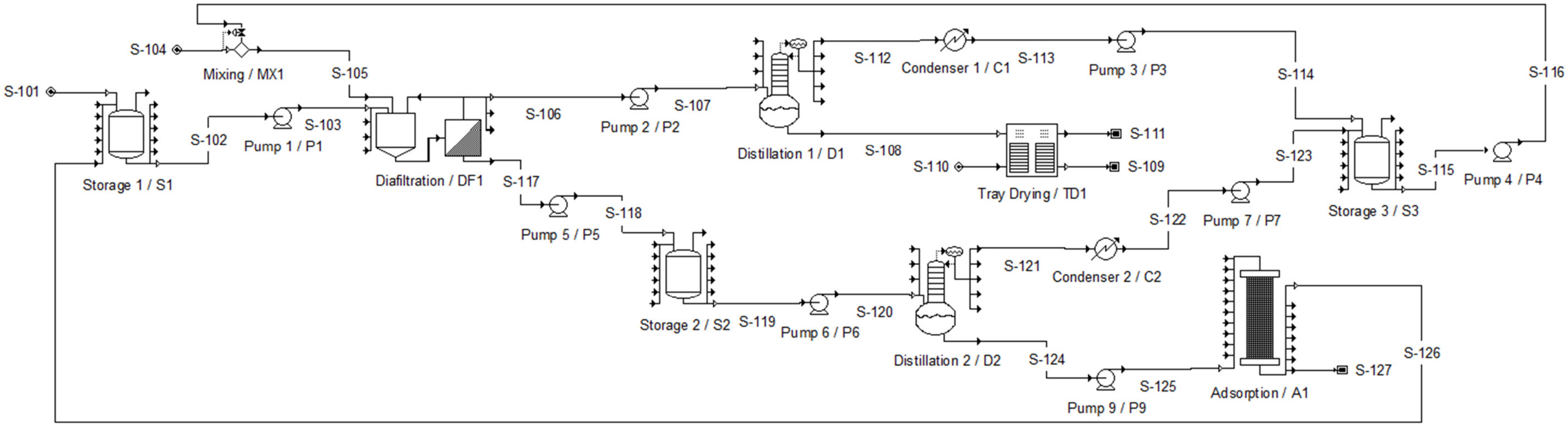

Figure S4. Process flow diagrams for the hybrid process using both organic solvent (dia)nanofiltration and column chromatography steps.

4.2. Economic Analysis Assumptions and Inputs - Additional Details

The following details were taken into consideration on the economic analysis:

• **Capital costs** include direct capital costs calculated considering equipment cost, while indirect capital costs were estimated using percentages of equipment cost for each section: 40% for equipment assembly, 70% for piping, 20% for instrumentation, 10% for electrical wiring, 15% for process building, 50% for utilities, 15% for storage, 5% for site development, 30% for design and engineering, 15% for contractors fee, and 10% for contingency [3].

• **Operational costs** were obtained using percentages of the total capital costs: 5% for maintenance, 20% for laboratory costs, 20% for supervision, 50% for plant overheads, 10% for capital charges, 1% for insurance, 2% for local taxes, and 1% for licence fees [3]. A 10-year period was considered for the economic analysis and amortization. Maintenance is dependent on direct capital cost, since it was calculated as a factor of 5% of capital costs.

• **Labour cost** was calculated using full time equivalent (FTE), times the number of workdays per year, multiplying by the number of batches per year and the wages. Labour cost for supervisors was considered as 40% of labour cost for operators.

• **Solvents:** Fresh dichloromethane used for 5% solvent make-up was determined to cost 0.9975 €/L.

• **Selective agent:** The price of the adsorber (PBI-TA and PBI-TB) was estimated to be 580 €/kg, accounting for commercial price of PBI at 336.80€/kg and thermal and acid or basic treatment. Membrane price was set at 8000€ per spiral wound module, each module having a membrane area of 6.5 m^2. Waste disposal was set at 0.5 €/kg [4].

• **Energy and utilities:** Cost of utilities was determined using the power multiplied by the working time of equipment (pumping); heating and cooling were determined through mass and energy balances.

4.3 Equipment Cost for Adsorption, OSN and Hybrid Processes

Table S8. Equipment cost for adsorption, OSN and hybrid processes.

Process	Equipment	Number of Units	Unitary Cost €	Total Cost €
Adsorption (DMAP)	Storage vessel 1 m^3	1	10,000	10,000
	Centrifugal pump	4	2500	10,000
	Chromatographic column 0.66 m^3	1	20,000	20,000
	Storage vessel 3 m^3	2	25,000	50,000
	Distillation column	1	75,000	75,000
	Condenser	1	25,000	25,000
	Boiler	1	20,000	20,000
	Dryer (tray)	1	100,000	100,000
	Total process			310,000
OSN (DMAP)	Storage vessel 1 m^3	1	10,000	10,000
	Centrifugal pump	7	2500	17,500
	Diafiltration (housing+pump)	1	30,000	30,000
	Storage vessel 3 m^3	2	25,000	50,000
	Distillation column	2	75,000	150,000
	Condenser	2	25,000	50,000
	Boiler	2	20,000	40,000
	Dryer (tray)	1	100,000	100,000
	Total process			447,500
OSN (MPTS)	Storage vessel 1 m^3	1	10,000	10,000
	Centrifugal pump	7	2500	17,500
	Diafiltration (housing+pump)	1	30,000	30,000
	Storage vessel 2.5 m^3	2	22,410	44,820
	Distillation column	2	75,000	150,000
	Condenser	2	25,000	50,000
	Boiler	2	20,000	40,000
	Dryer (tray)	1	100,000	100,000
	Total process			442,320
Hybrid (MPTS)	Storage vessel 1 m^3	1	10,000	10,000
	Centrifugal pump	8	2500	20,000
	Diafiltration (housing+pump)	1	30,000	30,000
	Storage vessel 3 m^3	2	25,000	50000
	Distillation column	2	75,000	150,000
	Condenser	2	25,000	50,000
	Chromatographic column 0.66 m^3	1	20,000	20,000
	Boiler	2	20,000	40,000
	Dryer (tray)	1	100,000	100,000
	Total process			470,000

4.4. Full Time Equivalent (FTE) for Each Process and Corresponding Labour Costs

Table S9. Full time equivalent (FTE) for each process and corresponding labour costs.

Process	Operation Time (h)	FTE	Cost (€/year)
Adsorption (DMAP)	8.3	1.0	12000
OSN (DMAP)	12.7	1.6	18000
OSN (MPTS)	12.1	1.5	17000
Hybrid (MPTS)	36.7	4.6	52000

4.5. Membrane Area Requirements and Corresponding Replacement Cost

Table S10. Membrane area requirements and corresponding replacement cost.

Process	Calculated Area (m^2)	Number of Modules Used and Area	Diafiltration Time Used (h)	Cost (€)
OSN (DMAP)	21.4	4 (4 × 6.5 m^2)	1.1	32,000
OSN (MPTS)	14.6	3 (3 × 6.5 m^2)	1.0	24,000
Hybrid (MPTS)	19	3 (3 × 6.5 m^2)	1.3	24,000

Note: Membrane area was calculated based on a fixed diafiltration time of 1.3 h.

References

1. Ferreira, F. A., Esteves, T., Carrasco, M. P., Bandarra, J., Afonso, C. A. M., Ferreira, F. C. Polybenzimidazole for Active Pharmaceutical Ingredient Purification: The Mometasone Furoate Case Study. Ind. Eng. Chem. Res. 2019, 58, 24, 10524-10532.
2. Székely, G.; Bandarra, J.; Heggie, W.; Ferreira, F. C.; Sellergren, B. Organic solvent nanofiltration: A platform for removal of genotoxins from active pharmaceutical ingredients. J. Memb. Sci. 2011, 381, 21-33.
3. Sinnott, R. K. *Chemical Engineering Design*, 4th ed., v 6, Butterworth-Heinenmann, 2005.
4. McKendry, Peter. *Costs of Incineration and Non-Incineration Energy from Waste Technologies*. Greater London Authority. 2008.
